# Organic substrate diffusibility governs microbial community composition, nutrient removal performance and kinetics of granulation of aerobic granular sludge

**DOI:** 10.1016/j.wroa.2019.100033

**Published:** 2019-05-20

**Authors:** M. Layer, A. Adler, E. Reynaert, A. Hernandez, M. Pagni, E. Morgenroth, C. Holliger, N. Derlon

**Affiliations:** a Eawag, Swiss Federal Institute of Aquatic Science and Technology, 8600, Dübendorf, Switzerland; b ETH Zürich, Institute of Environmental Engineering, 8093, Zürich, Switzerland; c Ecole Polytechnique Fédérale de Lausanne (EPFL), ENAC IIE Laboratory for Environmental Biotechnology, 1015, Lausanne, Switzerland; d SIB Swiss Institute of Bioinformatics, 1015, Lausanne, Switzerland

**Keywords:** Aerobic granular sludge, Influent composition, Low-strength municipal wastewater, Microbial community, Particulate substrate

## Abstract

Basic understanding of formation of aerobic granular sludge (AGS) has mainly been derived from lab-scale systems with simple influents containing only highly diffusible volatile fatty acids (VFA) as organic substrate. This study compares start-up of AGS systems fed by different synthetic and municipal wastewaters (WW), characterised by increasing complexity in terms of non-diffusible organic substrate. Four AGS reactors were started with the same inoculum activated sludge and operated for one year. The development of AGS, settling characteristics, nutrient and substrate removal performance as well as microbial community composition were monitored. Our results indicate that the higher the content of diffusible organic substrate in the WW, the faster the formation of AGS. The presence of non-diffusible organic substrate in the influent WW led to the formation of small granules and to the presence of 20–40% (% of total suspended solids) of flocs in the AGS. When AGS was fed with complex influent WW, the classical phosphorus and glycogen accumulating organisms (PAO, GAO) were outcompeted by their fermentative equivalents. Substrate and nutrient removal was observed in all reactors, despite the difference in physical and settling properties of the AGS, but the levels of P and N removal depended on the influent carbon composition. Mechanistically, our results indicate that increased levels of non-diffusible organic substrate in the influent lower the potential for microbial growth deep inside the granules. Additionally, non-diffusible organic substrates give a competitive advantage to the main opponents of AGS formation – ordinary heterotrophic organisms (OHO). Both of these mechanisms are suspected to limit AGS formation. The presented study has relevant implications for both practice and research. Start-up duration of AGS systems treating high complexity WW were one order of magnitude higher than a typical lab-scale system treating VFA-rich synthetic WW, and biomass as flocs persisted as a significant fraction. Finally, the complex synthetic influent WW – composed of VFA, soluble fermentable and particulate substrate - tested here seems to be a more adequate surrogate of real municipal WW for laboratory studies than 100%-VFA WW.

## Highlights

• Formation of aerobic granules with synthetic and real wastewaters was compared.
• Slower granulation with wastewaters containing high non-diffusible substrate.
• Larger floc fractions in biomass with non-diffusible substrate in influent.
• Similar microbial communities with different AGS characteristics.
• Complex synthetic wastewater can be a good surrogate for real wastewater.

## Graphical abstract

**Figure:**
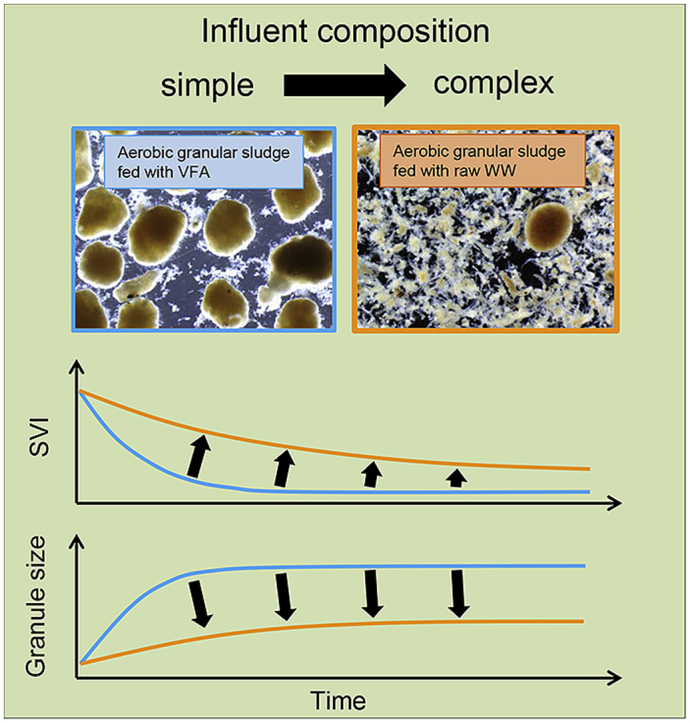

## 1 Introduction

Aerobic granular sludge (AGS) systems have been developed over the past 20 years and now offer a relevant alternative over conventional activated sludge systems (Morgenroth et al., 1997). Advantages of AGS include enhanced settling properties, a high suspended solid concentration and the co-existence of different redox conditions across the granules, which result in significant energy, footprint and chemical savings (Khan et al., 2015). Worldwide, more than 40 full-scale plants are now in operation, treating a wide range of municipal and industrial wastewaters (Pronk et al., 2017). However, the performance and/or granulation process of AGS systems are often hampered by the wastewater composition (Guimarães et al., 2018; de Kreuk and VAN Loosdrecht, 2006; Guimarães et al., 2017). To optimise the performances of such systems, it is therefore required to understand the link between the influent composition and the granule formation.

Lab-scale sequencing batch reactors (SBRs) have been extensively used to develop our fundamental understanding of AGS systems (Weissbrodt et al., 2013; He et al., 2016b; de Kreuk and VAN Loosdrecht, 2004). Those studies were mainly conducted using high concentrations of volatile fatty acids (VFA) (*e.g.*, acetate and/or propionate) and phosphorus. The key role of anaerobic feast and aerobic famine conditions on the granule formation was identified (de Kreuk and VAN Loosdrecht, 2004). These conditions favour the growth of slow-growing organisms like polyphosphate- (PAO) and glycogen-accumulating organisms (GAO), which have been identified as key players in granulation (de Kreuk and VAN Loosdrecht, 2004). The growth of PAO and GAO, and ultimately the granulation, is improved by the presence of soluble organic carbon (fermented or not) in the influent. The selective uptake of soluble organic carbon by PAO and GAO outcompete ordinary heterotrophic organisms (OHO). OHO growth hampers the formation of granular biomass or the nutrient-removal performances, while it also promotes the formation of flocs (de Kreuk et al., 2010; Pronk et al., 2015a; Weissbrodt et al., 2014; Novák et al., 1993). If the growth of PAO and GAO is crucial for the formation of aerobic granules during treatment of VFA-rich influent, it is then intuitive that granulation might be hampered during treatment of municipal WW containing high particulate organic substrate (X_B_) and low VFA fractions.

A key aspect in understanding AGS systems is in characterizing the microbial community composition and understanding how it influences the granulation process. The microbial communities of AGS systems fed with 100%-VFA WW are well described in literature, and dominated by Gammapropteobacteria, in particular, the PAO *Candidatus* (*Ca.)* Accumulibacter and the GAOs from the Competibacteraceae family (Weissbrodt et al., 2013; He et al., 2016a; Henriet et al., 2016). Most of these bacteria have a metabolism adapted to VFA uptake under anaerobic conditions. It is also likely that those bacteria are not able to ferment most sugars and amino acids or to hydrolyse polymers (Marques et al., 2017; Kong et al., 2006). So far, only few studies have characterised the microbial communities of AGS treating WW containing X_B_. Hence, the core microbial community of these AGS has not been identified yet (Wang et al., 2018; Szabó et al., 2017a; Świątczak and Cydzik-Kwiatkowska, 2018; Kang et al., 2018). Fermentative and hydrolysing bacteria are expected to be abundant in such systems, similarly to enhanced biological phosphorus removal (EBPR) systems treating municipal WW (Kong et al., 2008). The fermentative PAO *Tetrasphaera* does not store VFA in the form of polyhydroxyalkanoate (PHA) and is usually more abundant than *Ca.* Accumulibacter in Danish EBPR WW treatment plants (Mielczarek et al., 2013). *Tetrasphaera* can take up orthophosphate aerobically after anaerobic storage of different carbon sources like amino acids and glucose (Nguyen et al., 2011). *Micropruina* is also commonly found in EBPR activated sludge (Saunders et al., 2016; Stokholm-Bjerregaard et al., 2017). *Micropruina* is a fermentative GAO able to take up and ferment various carbon sources anaerobically to constitute glycogen reserves (Mcilroy et al., 2018). It is however unclear to what extent *Tetrasphaera* and *Micropruina* play a role in the formation of AGS during treatment of complex WW with a high X_B_ content. A key aspect of this study will be to characterise the microbial communities found in AGS systems fed with WW containing different fractions (and types) of X_B_. Another objective will be to identify correlations between these communities and the sludge settling properties and nutrient removal performance.

If the WW composition influences the microbial community, it is reasonable to expect that the granulation process of AGS systems is also impacted (*e.g.*, physical properties of biomass and start-up kinetics). A harsh selection of fast settling biomass in lab-scale reactors fed with 100%-VFA synthetic WW resulted in rapid granulation within two weeks (Weissbrodt et al., 2013; Mosquera-Corral et al., 2011). But nutrient-removal was impaired for weeks to months. Lochmatter and Holliger (2014) successfully started up an AGS system within 28 days without loss of nutrient removal, by applying a more gentle washout of slow settling biomass and by adapting the organic loading during the early stages of start-up. Start-up of AGS systems with municipal WW can be significantly longer. Harsh selection pressure on slow-settling biomass can lead to fast granulation (*e.g.*, 20 days, de Kreuk and VAN Loosdrecht, 2006) but the time reported to transform activated sludge into AGS while maintaining the nutrient-removal performance have normally been much longer (40–400 days) (Liu et al., 2010; Giesen et al., 2013; Derlon et al., 2016). Therefore, this study aims to clarify the link between start-up kinetics and influent WW composition, while similar operating conditions are applied and the same inoculum activated sludge is used.

**Fig. 1:**
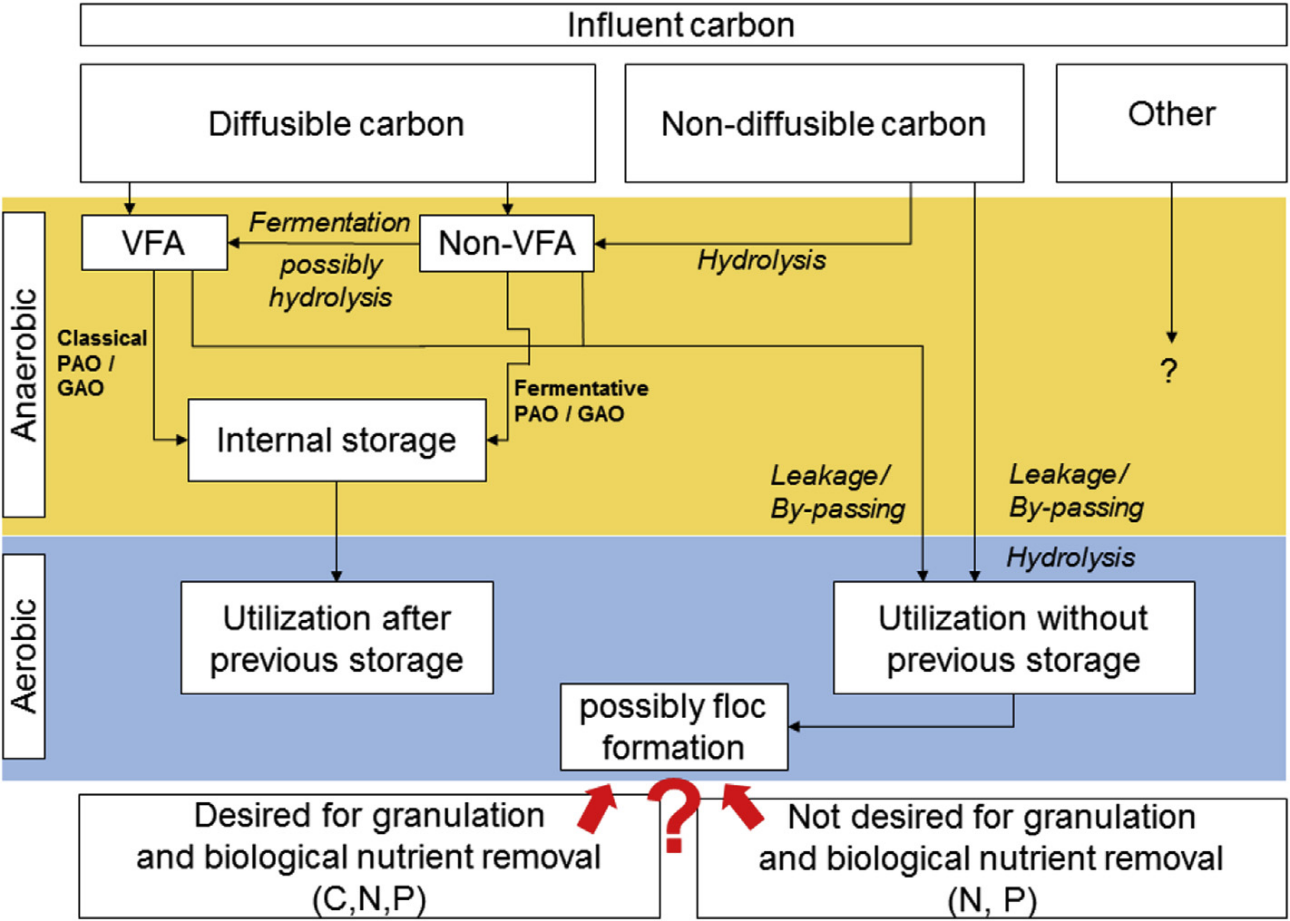
Conceptual model of carbon utilization and proposed desired/undesired pathways in AGS systems, given plug-flow anaerobic feeding and subsequent aerobic fully mixed conditions.

The diffusibility and uptake rate of organic carbon directly influences the microbial competition for substrate, and in turn the granulation (Fig. 1). A slow anaerobic conversion of non-diffusible X_B_ combined with a decreased substrate availability within the granule can result in carbon leakage (i.e., carbon available in aerobic conditions). Carbon leakage favours OHO growth to the detriment of PAOs, GAOs and fermenters, and ultimately result in floc formation (Larsen and Harremoës, 1994; Morgenroth et al., 2002; Wagner et al., 2015; Jabari et al., 2016; Suresh et al., 2018). For municipal WW, non-diffusible X_B_ usually represents 50% of the total influent chemical oxygen demand (COD) (Tchobanoglous et al., 2014). Based on the current knowledge, the formation of AGS during treatment of X_B_ rich WW might thus be hampered. In addition, it remains unclear whether flocs are detrimental to AGS systems, when non-diffusible X_B_ represents a high proportion of the influent COD.

The main goal of this study was to understand the link between influent WW composition, microbial community, physical AGS parameters and nutrient removal performance. The specific research questions were to better understand how the WW composition, in terms of diffusible and non-diffusible organic substrates, (i) influences the overall microbial community development, (ii) divides the microbial community between flocs and granules, (iii) governs nutrient removal, (iv) defines physical characteristics such as settling properties, sludge morphology, and (v) influences the success and duration of start-up of AGS systems when similar operating conditions are applied. Four lab-scale SBR were inoculated with the same activated sludge and operated for over 400 days in parallel. Four distinct WWs were used: 100%-VFA synthetic, complex synthetic, municipal primary effluent, and municipal raw WW. The sludge properties (morphology, concentration, SVI, and size distribution), reactor performances (C, N, P and total suspended solids (TSS) removal), and microbial community composition of the flocs and granules were monitored by 16S rRNA amplicon sequencing.

## 2 Materials and methods

### 2.1 Experimental approach

Four SBRs were operated in parallel for 400 days and fed with four different WWs: 100%-VFA (acetate, propionate) synthetic WW (R1), complex synthetic WW (R2), primary effluent municipal WW (R3), and raw municipal WW (R4). Those four WWs mainly differed with regards of the carbon source, *i.e.*, concentrations in volatile fatty acids, soluble and particulate organic substrates (Table 1). After approximatively three months of operation, R4 was restarted due to complete sludge loss. Data of R4 (fed with raw WW) are thus shown for the first run (run#1) and the second run (run#2).

**Table 1:** Measured influent composition of the four SBRs fed by 100%-VFA synthetic WW, complex synthetic WW, primary effluent WW and raw WW, specific substrate recipe of R1 and R2 influent are given in Supplementary Information Table S2.^a^

Reactor	100%-VFA synthetic WW	complex synthetic WW	primary effluent WW	raw WW	raw WW
	R1	R2	R3	R4 run#1	R4 run#2
Total COD [mg COD L^−1^]	582 ± 65	503 ± 61	331 ± 97	808 ± 42	469 ± 151
Soluble COD [mg COD L^−1^]	582 ± 65	457 ± 73	188 ± 76	271 ± 109	247 ± 121
Particulate COD [mg COD L^−1^]	0 ± 92	46 ± 95	143 ± 123	537 ± 441	222 ± 194
VFA [mg COD L^−1^]	582 ± 65^b^	170 ± 26^b^	26 ± 17^c^	-d	40 ± 28^e^
Ac + Pr [mg COD L^−1^]	582 ± 65	170 ± 26	15 ± 9	–	17 ± 11
Ac + Pr/total COD-ratio	1.00	0.33	0.05	–	0.06
Total nitrogen (TN) [mg N L^−1^]	43 ± 10	44±	33 ± 9	30 ± 7	41 ± 19
NH_4_–N [mg N L^−1^]	40 ± 8	20 ± 5	24 ± 6	25 ± 4	29 ± 10
Total phosphorus (TP) [mg P L^−1^]	5.4 ± 0.9	5.4 ± 1.7	3.3 ± 0.9	3.2 ± 0.5	4.4 ± 1.9
PO_4_–P [mg P L^−1^]	5.0 ± 1.1	4.7 ± 0.8	2.3 ± 0.5	2.6 ± 0.4	2.7 ± 0.8

a Average and standard deviation (SD) were calculated from 24 to 38 measurements for R1, R2, R3, 13–15 for R4 run#1 and 13–22 for R4 run#2, respectively.

b VFA composition of synthetic WW: 50% Acetate, 50% Propionate (COD based).

c VFA composition of municipal primary effluent WW: 16% Acetate, 41% Propionate, 43% longer-chained VFAs (COD based).

d VFA composition of municipal raw WW run#1 was not measured.

e VFA composition of municipal raw WW run#2: 13% Acetate, 52% Propionate, 35% longer-chained VFAs (COD based).

### 2.2 Experimental set-up

The four SBRs comprised a mixed liquor volume of 12.9 L (height-to-diameter ratio 8.4), and were operated in simultaneous fill-draw mode. The SBR cycles consisted of the following phases: (i) anaerobic phase (90 min), (ii) aerobic phase (240 min), (iii) settling (duration see description below) and (iv) selective excess sludge withdrawal (60 s), with a total cycle length of 5.6 h (4.3 cycles per day). The anaerobic phase comprised an anaerobic plug-flow feeding (PF) and an anaerobic idle. The latter was changed to anaerobic mixing on day 357 and 289 for reactors R3 and R4 run #2, respectively. The WW upflow velocity during PF feeding (v_ww_) was set to 0.25 m h^−1^ (anaerobic PF + idle) and 0.38 m h^−1^ (anaerobic PF + mixing). The volume exchange ratio (VER) was set to 0.3. The oxygen concentration during the aerobic phase was controlled with a setpoint of 2.00 mg O_2_ L^−1^. Mixing was provided by a mechanical stirrer during anaerobic PF + mixing, and by aeration during aerobic conditions. All SBRs were equipped with oxygen sensors (Optical LDO, Endress & Hauser, Switzerland). Both sensors were connected to a programmable logic controller (PLC), which was controlled and monitored by a supervisory control and data acquisition (SCADA) system. All reactors were inoculated with activated sludge from the WW treatment plant (WWTP) Thunersee, Switzerland, which performs biological carbon, nitrogen and phosphorus removal.

### 2.3 Start-up approach

The start-up approach was based on the strategy developed by Lochmatter and Holliger (2014). The selective pressure on slow settling biomass was first maintained at a low level, in order to prevent too high washout stress. This was achieved by slowly increasing the critical settling velocity (v_crit_) from 1.7 to 5.1 m h^−1^. An increase in v_crit_ was iteratively reassured by SRT calculations (Equation (1)).

(1) $$SRT=\;\frac{V_{r}\cdot TSS_{r}}{Q_{ex}\cdot TSS_{ex}+Q_{eff}\cdot TSS_{eff}}$$

V_r_ is the reactor volume (L), TSS_r_ is the TSS concentration in the reactor (gTSS L^−1^), Q_ex_ is the flow rate of excess sludge (L d^−1^), TSS_ex_ is the TSS concentration of the excess sludge (gTSS L^−1^), Q_eff_ is the flow rate of effluent (L d^−1^), and TSS_eff_ is the TSS concentration in the effluent (gTSS L^−1^). If SRT was <20 d, v_crit_ was decreased again. The procedure was repeated on a weekly basis. Also, long anaerobic and aerobic phases were applied (total cycle duration 5.6 h), in order to improve anaerobic COD uptake and aerobic nutrient removal. Finally, v_ww_ was kept low (0.25–0.38 m h^−1^), in order to provide a high substrate gradient during anaerobic plug-flow feeding and improve anaerobic COD uptake.

### 2.4 Start-up definition

We here define successful start-up of AGS on both physical properties and substrate/nutrient removal. Specifically, the settling parameters SVI_30_ < 90 mL g^−1^ and SVI_30/10_-ratio > 0.8, the size fraction d > 0.25 mm constituting at least 50% of TSS, granule appearance based on microscopic images, and stable substrate and nutrient removal. Parameters and values were selected based on previous experience on the start-up of AGS systems for the treatment of low strength municipal WW at Eawag (Wagner et al., 2015; Derlon et al., 2016) and by other researchers/practitioners (*e.g.*, Ni et al., 2009; Liu et al., 2011; Giesen et al., 2013; van der Roest et al., 2011; Coma et al., 2012; de Kreuk and VAN Loosdrecht, 2006; Pronk et al., 2015b). The definition is in line with the original definition of AGS (de Kreuk et al., 2007).

### 2.5 Wastewater composition and sludge inoculum

The detailed influent composition of R1, R2, R3 and R4 are shown in Table 1. All WW were in the range typical of low to medium strength WW (Tchobanoglous et al., 2014). Synthetic substrates comprised a total carbon:nitrogen:phosphorus ratio of approx. 100:7:1. Acetate (Ac) and propionate (Pr) were used as sole carbon source for the 100%-VFA synthetic WW (50% of COD each). Complex synthetic WW was composed of 1/3 VFAs (1/6 acetate + 1/6 propionate), 1/3 soluble fermentable substrates (1/6 glucose, 1/6 amino acids) and 1/3 particulate substrates (1/6 peptone, 1/6 starch). Particulate substrates were peptone from gelatin, enzymatic digest (Fluka Analytical, Switzerland), and starch made from wheat (Merck KGaA, Germany). Amino acids were composed of l-alanine, l-arginine, l-aspartic acid, l-glutamic acid, l-leucine, l-proline and glycine in equal COD-equivalents. These individual amino acids were chosen according to the most abundant amino acids present in the peptone used. Added nitrogen was composed of soluble NH_4_–N for the system with 100%-VFA synthetic WW but included nitrogen from peptone and amino acids for complex synthetic WW. Phosphorus was composed of soluble PO_4_–P species for both synthetic WWs. In order to prevent bacterial growth in the synthetic substrate storage bottles, the phosphorus species and diluent water were stored separately from the nitrogen and carbonaceous species and mixed automatically before each cycle (Ebrahimi et al., 2010). The nitrogen and carbonaceous species were prepared in 20-fold concentration in portions of 5 L and, after addition of 50 mL of a trace-element solution (Supplementary Information Table S1). Municipal WW from the city of Dübendorf, Switzerland, was used. Effluents of grit and fat removal (raw WW) and additional primary clarification (primary effluent WW) from the pilot-scale WWTP at Eawag were used for this study.

### 2.6 Physical sludge parameters

TSS, VSS and SVI_5_, SVI_10_, SVI_30_ were quantified using standard methods (APHA, 2005). Additionally, the SVI_30/10_ and SVI_30/5_ ratios were calculated. The sludge size fractions were separated by sieving the sludge at 1, 0.63 and 0.25 mm, respectively. Granules were associated with fractions d > 0.25 mm, flocs d < 0.25 mm. Size fractions were then quantified based on TSS measurements. Sludge morphology was observed by stereomicroscopy (Olympus, SZX10, Japan) on weekly – bi-weekly basis.

### 2.7 Analytical methods

Samples of influent and effluent were analysed for COD, total nitrogen (TN) and total phosphorus (TP) using photochemical tests (Hach Lange, Germany, LCK 114, 314, 338, 238, 348, 349). Soluble COD (sCOD) was measured after filtration at 0.45 μm (Macherey Nagel, Nanocolor Chromafil membranefilter GF/PET 0.45 μm, Germany). Cations (NH_4_^+^-N) and anions (NO_3_^−^-N, NO_2_^−^-N, PO_4_^3-^-P) were measured using flow injection analysis (Foss, FIAstar flow injection 5000 analyzer, Denmark) and anion chromatography (Methrom, 881 compact IC, Switzerland), respectively. VFAs were measured using headspace solid-phase microextraction (HS-SPME) followed by gas chromatography coupled to flame ionization detection (GC-FID) (Trace 1300 GC, Thermo Scientific, USA) (Feng et al., 2008).

### 2.8 Microbial community analysis

#### 1.1.1 Biomass sampling

Both granules and flocs were collected for analysis of the microbial community composition after sieving at 250 μm. Biomass samples of around 1 mL were centrifuged (5 min,4500 rpm) (Nuaire Awel CF-48R centrifuge, U.S.A) then washed twice by addition of 5 mL of ice-cold phosphate buffer saline (PBS) solution and then centrifuged again (5 min, 4500 rpm). Pellets were then re-suspended in 3 mL of PBS solution, homogenized with a glass homogenizer, distributed in cryotubes and stored at −80 °C until DNA extraction. 200 μL of homogenized biomass were mixed with 400 μL of elution buffer (T_10_E_0.1_) and 100 μL of lysozyme solution (25 mg mL^−1^). After 1 h at 37 °C, DNA was extracted using an automatic robot 16 DNA Purification System (Maxwell, Promega Corporation, Switzerland). The DNA concentration of each DNA extraction was measured with a spectrophotometer NanoDrop ND1000 (Witec AG, Switzerland). The bacterial 16S rRNA gene hypervariable regions V1–V2 were amplified by polymerase chain reaction (PCR) in a T3000 Thermocycler (Biometra GmbH, Germany) using the universal primers 27F and 338R and the High-Fidelity Q5 polymerase (High-fidelity 2x Master Mix, Biolabs Inc., USA), according to the protocol in supplementary information S3. The amplified DNA was quantified using the DNF-473 standard sensitivity NGS fragment analysis kit (Advanced Analytical Technologies Inc., U.S.A). The Lausanne Genomic Technologies Facility (University of Lausanne, Switzerland) performed secondary indexing PCR and multiplex sequencing by groups of 96 samples per run on an Illumina MiSeq platform in paired-end mode (2x250). The sequences were deposited at the European Nucleotide Archive (ENA) under the study accession number ERP111727.

#### 1.1.2 Taxonomic affiliation of 16S rRNA gene sequences

The amplicon sequences were demultiplexed and primers removed. The trimming and quality filtering of the sequences was performed using trimmomatic v.0.36 (Bolger et al., 2014) with a sliding window of 4 base pairs (bp), a quality score threshold of 15 and a minimal length of 100 bp. The paired-end reads were merged with Pear v 0.9.11 (Zhang et al., 2014). The sequences were then grouped with a minimum similarity threshold of 97% using the clustering software cd-hit v.4.6.1 (Fu et al., 2012). Clusters with less than 5 sequences per sample on average were discarded. The cluster heads of the remaining clusters were compared with the 16S rRNA gene database MiDAS v.S123_2.1.3 (Mcilroy et al., 2015) using the blast software (Altschul et al., 1990). For each cluster, the taxonomy of the best match with the cluster head was attributed to all the sequences of the cluster. The level of precision of the taxonomy was adjusted according to the percentage of similarity with the threshold sequence identity values given by (Yarza et al., 2014), 94.5% for genus, 86.5% for family, 82.0% for order, 78.5% for class and 75.0% for phylum. For example, if a sequence had 90% of similarity with its best match, the taxonomy attributed to its cluster was precise only up to the family level.

#### 1.1.3 Statistical analysis

All the statistical analyses and the related plots were performed with R program v.3.5.0 (R Core Team, 2018) using the packages reshape2, gplots and ggplot2 (Wickham, 2007, 2016; Warnes et al., 2016). Bray-Curtis distance matrices, the associated principal coordinates analysis (PCoA) and Mantel-tests were done with the package vegan v.2.5–2 (Oksanen et al., 2018). A multifactorial analysis was performed with the R-package FactoMineR (Lê et al., 2008).

#### 1.1.4 Determination of the stable state and the discriminant taxa

After visual inspection of the PCoA plots on the Bray-Curtis distance matrix of the bacterial operational taxonomic units (OTU, 97%) relative abundance, the biomass communities of the different samples were separated in two states; a “transition state” and a “stable state”. For each reactor, a bacterial community was considered in the stable state if the maximum Bray-Curtis pairwise distance with the communities of all the following sample points was below 0.6 (the maximal distance between all the samples was 0.88). Five stable state datasets corresponding to the stable states of the 4 reactors and of the inoculum were analysed further. In order to extract the taxa that are discriminant between these five stable states, the mean relative abundances of the genera were compared two by two. After Hellinger transformations, the means were compared by using t-tests. The taxa are considered “divergent” if their mean in the stable dataset is significantly different (p-value 0.01 corrected for multiple testing using Bonferoni correction, p-value = 0.01/422 = 2.37 E−05) between at least two stable states. Taxa are considered “abundant” if their average abundance during stable state is higher than 1% in at least one stable dataset. The taxa being divergent and abundant are considered as “discriminant taxa” in the following analysis. There were 56 abundant, 273 divergent and 38 discriminant taxa on a total of 422 (at genus level).

#### 1.1.5 Comparison of the bacterial communities in flocs and granules

The average proportions of the most abundant genera were compared in each reactor with t-tests, in order to evaluate potential differences in the microbial communities in flocs and granules. The results with a p-value lower than the Bonferoni corrected p-value of 0.01 (p-value = 0.01/20 = 0.0005) were considered as significant.

## 3 Results

### 3.1 Settling properties

**Fig. 2:**
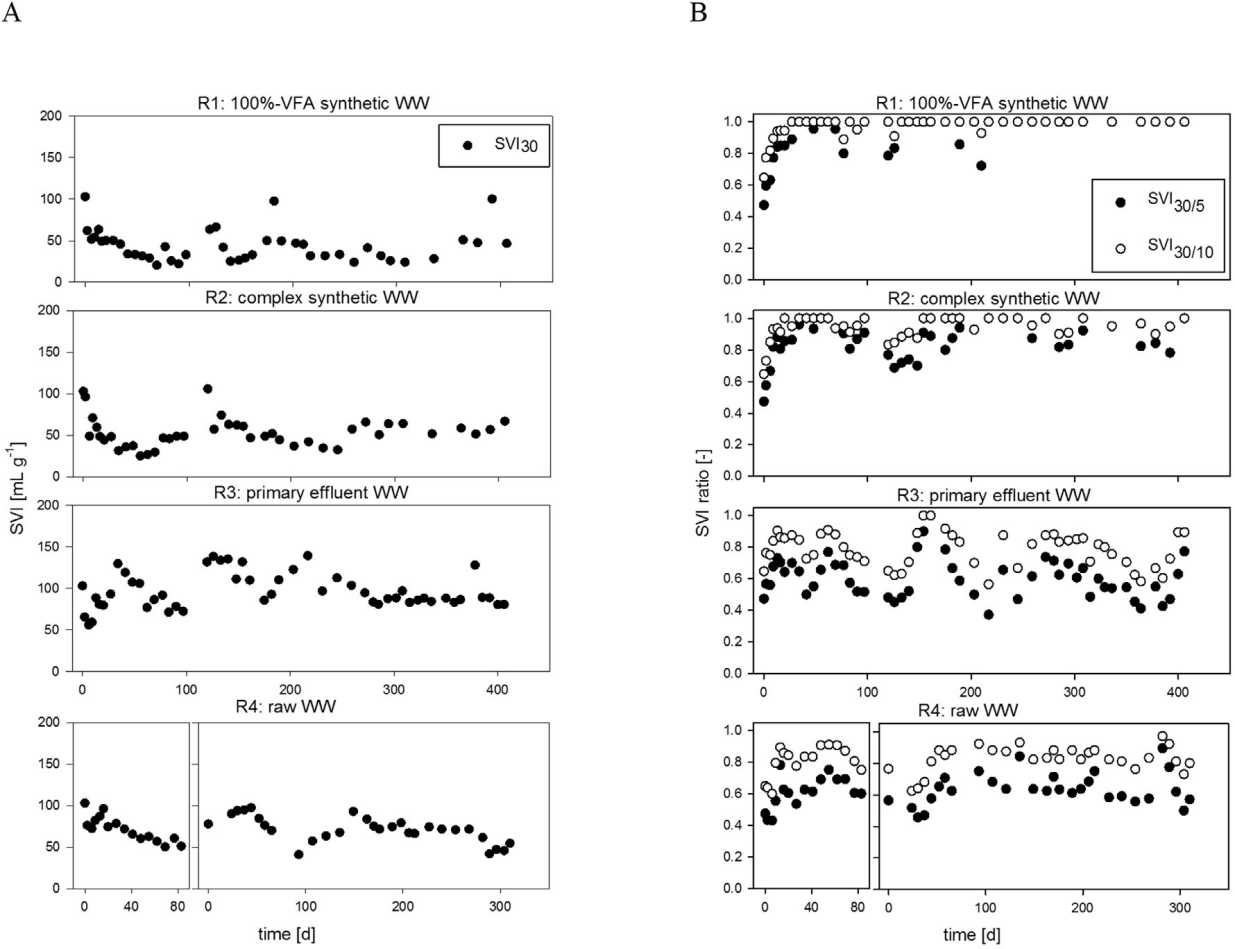
Evolution of (A) the sludge volume index SVI_30_ (measured after 30 min) and (B) the SVI ratios (30/5 and 30/10) of the aerobic granular sludge of the four SBRs. All SVI-values are provided in Supplementary Information Fig. S4.

A higher amount of diffusible organic substrate (VFA or fermentable) in the WW resulted in better settling properties (Fig. 2). Low SVI_30_ and SVI-ratios close to 1 were measured for the AGS of R1 and R2 (high content in diffusible organic substrate). Larger SVI_30_ and SVI-ratios close to 0.8 were on the other hand measured for the AGS of R3 and R4 (high content in non-diffusible organic substrate).

The SVI_30_ of the sludge of R1 and R2 decreased rapidly during the first 30 days of operation, and then stabilised at 30–50 mL g^−1^. Simultaneously, SVI-ratios > 0.9 were measured. Only 1–2 weeks were thus required to achieve granulation in these two reactors based on their SVI_30_ values. Over long term, the SVI_30_ and SVI ratios of AGS of R2 remained more variable in comparison with the ones of R1.

Achieving good settling properties for the systems fed by municipal WW required a much longer period (several months to over 1 year). The SVI_30_ values of the AGS of R4 steadily decreased within the first 100 days of operation to 50 mL g^−1^ for both runs and stabilised at SVI_30_ < 70 mL g^−1^ after 170 d for R4 run#2. AGS of R3 responded sensitively to changing operating conditions, which resulted in variable and high SVI_30_ values from day 0–200. SVI_30_ < 80 mL g^−1^ and SVI_30/10_ ratio >0.8 were finally achieved quickly after introduction of anaerobic PF + mixing (from day 357) for R3.

Successful start-up based on settling parameters (SVI_30_ < 90 mL g^−1^ and SVI_30/10_ ratio > 0.8) was achieved within the first two weeks for R1 and R2. A much longer start-up time was required for AGS of R4 and R3. Around 34 and 163 days were required to achieve successful start-up based on settling parameters for AGS of R4 run#2 and R4 run#1, respectively. R3 was successfully started-up only after 400 days.

### 3.2 Sludge size fractions

**Fig. 3:**
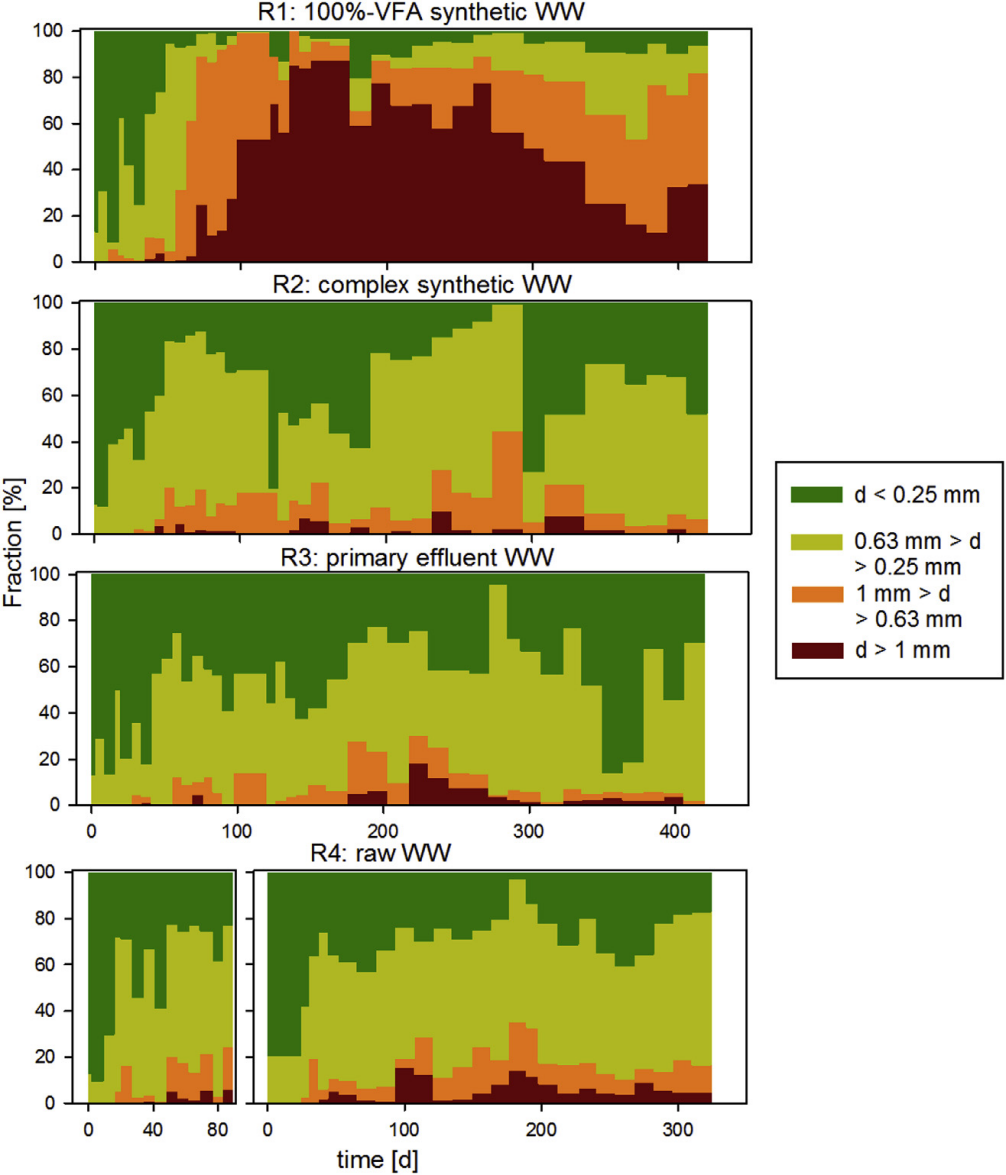
Evolution of the sludge size fractions (TSS based) for the aerobic granular sludge fed with different influent composition: 100%-VFA synthetic (R1), complex synthetic (R2), primary effluent (R3) and raw influent WW (R4) run#1 and run#2. Aggregates with d < 0.25 mm are considered as flocs, aggregates with d > 0.25 mm and <0.63 mm as small granules, aggregates with d > 0.63 mm and d < 1 mm as medium granules and aggregates with d > 1.0 mm as large granules.

The effect of the influent composition on the granulation process was confirmed by monitoring the different biomass size fractions (Fig. 3). The size of the granules greatly varied as a result of the presence of non-diffusible X_B_ in the influent. High fractions of large granules (d > 1 mm) were observed in R1 only, while smaller granules mixed with flocs were observed in R2, R3 and R4 (both runs). Overall, successful granulation based on size fractions (d > 0.25 mm of at least 50% of TSS) was achieved after 1–1.5 month of operation in all systems.

AGS of R1 was dominated by medium and large diameter granules (d > 0.63 mm), while flocs (d < 0.25 mm) represented only a minor fraction. The fraction of granules steadily increased in R1, while flocs simultaneously decreased to about 5% after 50 days of operation. Large granules gradually replaced small granules and dominated the sludge composition after 100 days of operation. The total fraction of granules (d > 0.25 mm) in R1 remained steady throughout the entire reactor operation, despite some fluctuations in the individual fractions. A major loss of large granules was observed after 300 days of operation in R1. Incomplete uptake of carbon during anaerobic conditions, possibly caused by-pass of the settled sludge bed during plug-flow feeding, resulted in filamentous outgrowth of the granules (Supplementary Information Fig. S5d). Filamentous outgrowth first resulted in an increase of sheared-off debris (indicated by an increase in d < 0.63 mm size fractions), followed by breakage of large granules. After 390 days of operation though, large granules started to develop again.

AGS fed with complex influent WW were mainly composed of small granules (50–70%) and flocs (20–40%). The AGS size fractions measured in the reactors fed with complex synthetic and municipal WW were very similar. Small granules represented the predominant size fraction, with 60–80%. Almost no large granules developed in these systems (rarely above 10% of total biomass). Also, the fractions of flocs decreased from 80% to less than 40% within the first 40 days. After day 40, 20–40% of flocs remained in the systems until the end of the experiment. Large fluctuations of size fractions – mainly of flocs - were only observed in R2 and R3.

### 3.3 Evolution of the bacterial community composition from inoculation to stable state

**Fig. 4:**
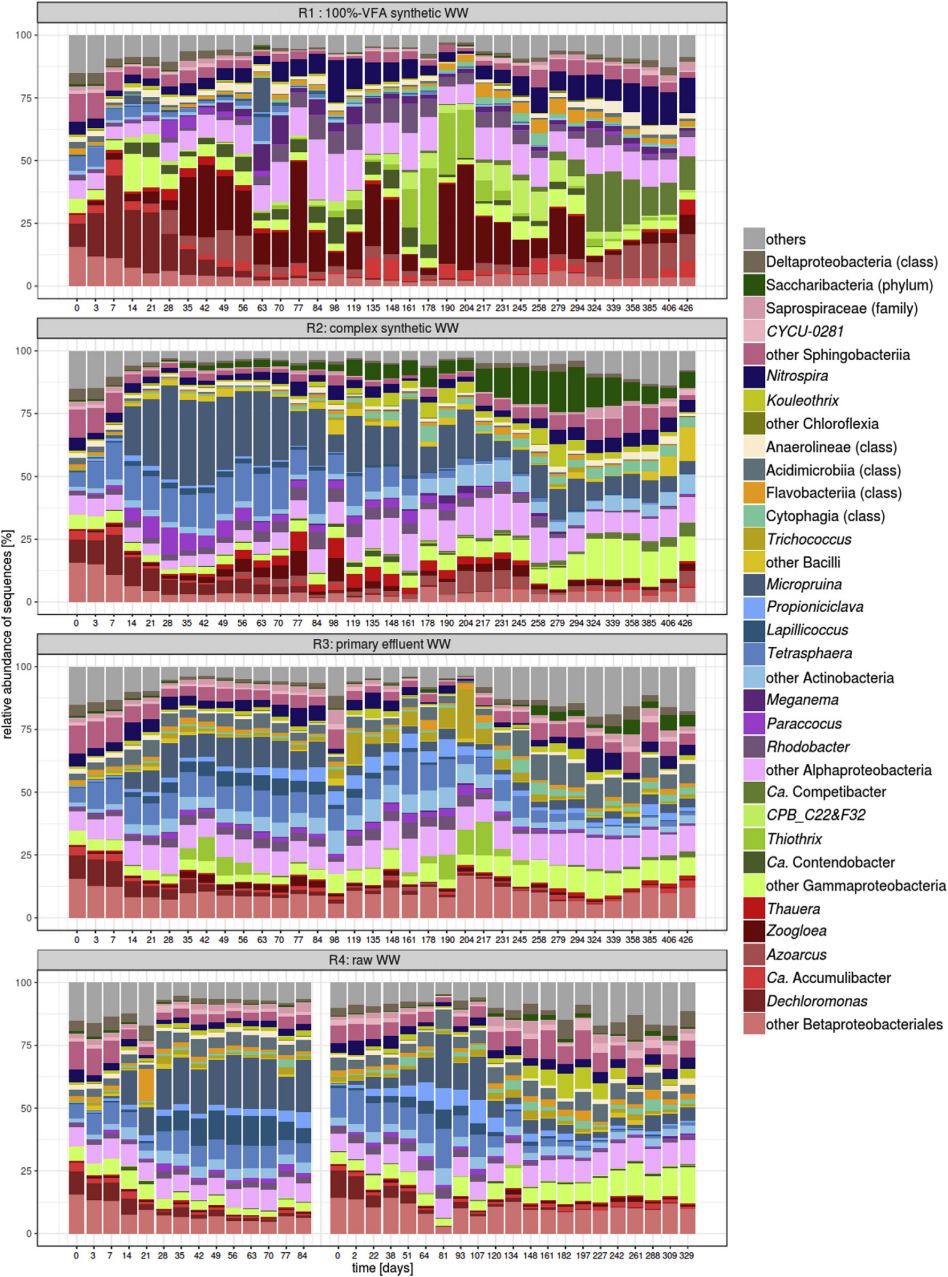
Composition of bacterial communities from inoculation to stable state in the four AGS reactors treating different types of WW. The most abundant taxa are shown in colors depending on the class they belong to. One exception is the order Betaproteobacteriales (colored in red) that has recently been included in the class Gammaproteobacteria (Parks et al., 2018). The other taxa of the latter class are colored in green. The evolution of the bacterial community of the reactor treating raw WW (R4) is shown for both run#1 and run#2. (For interpretation of the references to color in this figure legend, the reader is referred to the Web version of this article.)

The microbial communities were monitored over 426 days for the four reactors (267 samples, 12 millions of reads) (Fig. 4). The microbial communities developed differently in R1 (100%-VFA) than in R2, R3 and R4, with the latter two being very similar to each other. The microbial community in R2 (complex synthetic WW) was rather similar to the ones of R3 and R4, although some differences could be observed. In R1, an initial increase of the Gammaproteobacteria with successive changes within this class was observed. *Dechloromonas* was progressively replaced by other Betaproteobacteriales such as *Azoarcus* or *Zoogloea* whose relative abundance fluctuated greatly during the experiment. The proportion of Actinobacteria, here comprising mainly putative fermenting bacteria, gradually decreased to below 2% after day 119. *Tetrasphaera* and *Ca*. Accumulibacter represented 8% and 3% of the inocula communities, respectively. In R1, the abundance of *Tetrasphaera* decreased progressively and was <0.5% after 100 days whereas the abundance of *Ca.* Accumulibacter fluctuated between 0.1% and 8%. In R2, R3, and R4, Actinobacteria became abundant (30–50%) during the two first weeks of operation and stabilised at around 10% after 200 days of operation. The abundance of *Tetrasphaera* remained quite stable during the first 130 days in these reactors and then decreased to 1–3% whereas abundance of *Ca.* Accumulibacter was always <3%.

**Fig. 5:**
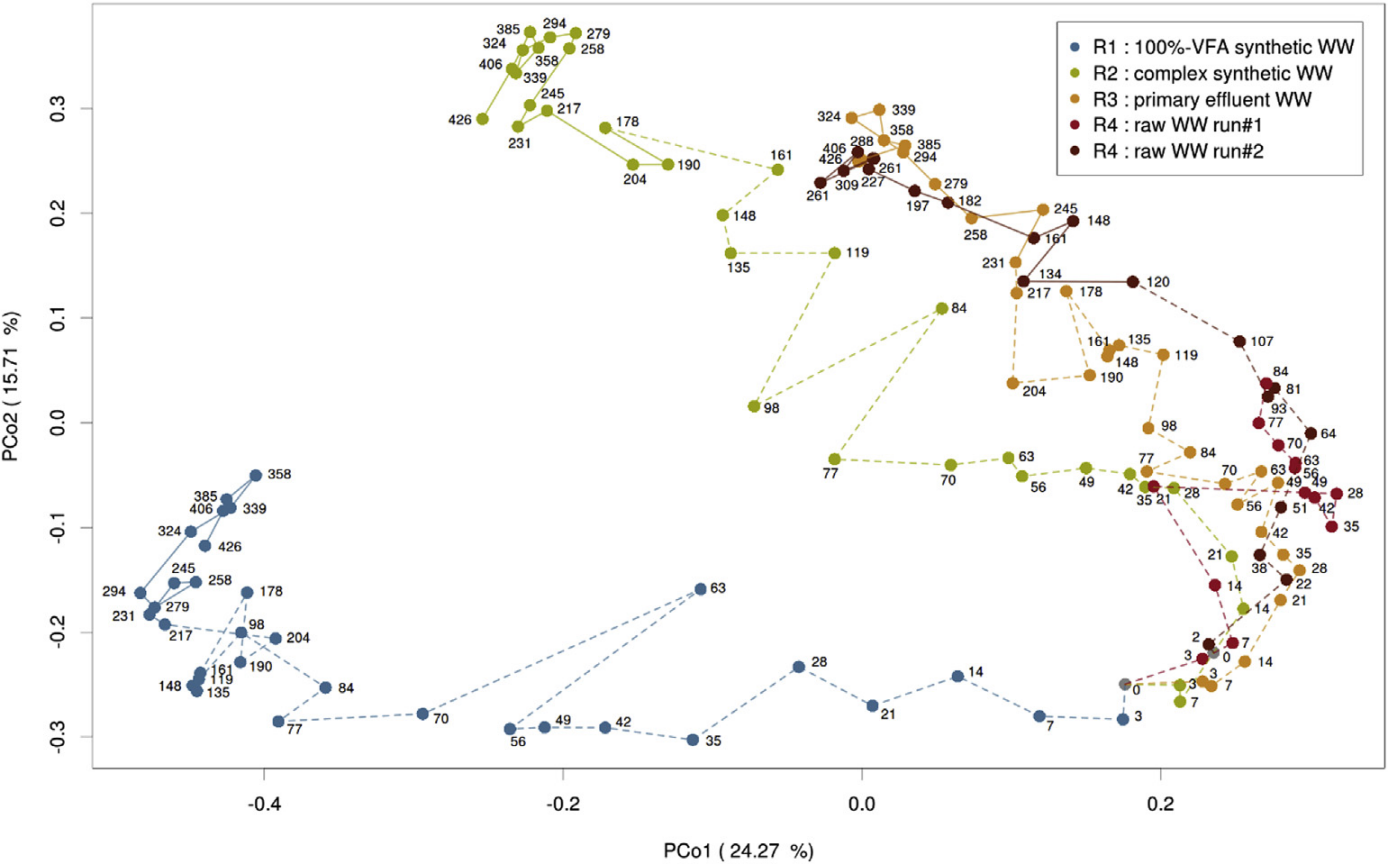
Principal coordinate (PCoA) plot based on the Bray-Curtis distance matrix of the bacterial OTUs relative abundance in the sludge samples collected in the four reactors. The samples are linked with dashed lines during the transition state and with solid lines during the stable state. Plots of PCo3 (explains 12.87% variance) vs PCo1 and PCo2 are provided in Supplementary Information Fig. S7.

The evolution of the microbial communities according to the different WWs are represented in the PCoA of the Bray-Curtis distance matrix of the bacterial OTUs (97%) relative abundance (Fig. 5). In all reactors, the bacterial community quickly changed first after inoculation (‘transition phase’) and then stabilised (‘stable state’). The bacterial communities of R3 and R4 (municipal WW) evolved towards a similar stable state. The bacterial community of R1 evolved very differently from R3 and R4. The evolution of the bacterial community of R2 was different from the one of R1 and quite close to the ones in reactors R3 and R4. The time to reach stable state significantly varied from one reactor to another. In R1 and R3 the bacterial community stabilised after 231 days, while 178 and 120 days were required for R2 and R4, respectively. The evolution of bacterial communities of R4 was similar during run#1 and run#2. The Shannon diversity index decreased during the transition phase, in particular for the reactors treating synthetic WW (Supplementary Information Fig. S8). The index was higher in the samples of the stable states, in comparison to the samples of the transition phases, which supports the pertinence of the criterion applied to determine the stable state.

A comparison of the proportions of each genus was performed in order to identify the taxa responsible for the differences between the different microbial communities of the four AGS systems and the inocula (Supplementary Information Fig. S11). Out of the total 422 taxa, 38 were identified as discriminant. Some of the discriminant taxa were characteristic for AGS fed with the simple WW (R1), such as *Meganema* or members of the Rhodobacteraceae family (Supplementary Information Fig. S11). Other taxa were mainly abundant in the AGS fed with real WW (inocula, R3 and R4), such as *Propioniciclava*, *Iamia*, *Acidovorax*, *Kouleothrix*, *Ca*. Epiflobacter and *Sulfuritalea*. Several taxa were more abundant in AGS treating complex WW, whether synthetic or municipal: *Microlunatus*, the fermentative GAO *Micropruina* or the fermentative PAO *Tetrasphaera*. The following genera were abundant and present in the ‘core community’ of the systems but not discriminant: the PAO *Ca.* Accumulibacter, the GAO *CPB_C22&F32*, *Ca*. Microthrix, Saprospiraceae (f), *Rhodobacter*, *Thauera*, and *Thiotrix*. Finally, the proportions of the bacterial taxa of R1 and R2 were compared separately, with those of R3 and R4 combined. This comparison was performed to assess the effect of synthetic vs. real WW on the microbial communities of AGS. Among the 29 abundant taxa in R3 or R4, 23 were found in significantly lower proportions in R1. In R2, the number of underrepresented taxa dropped to only 15 (Supplementary Information Table S9).

### 3.4 Bacterial communities of granules and flocs

**Fig. 6:**
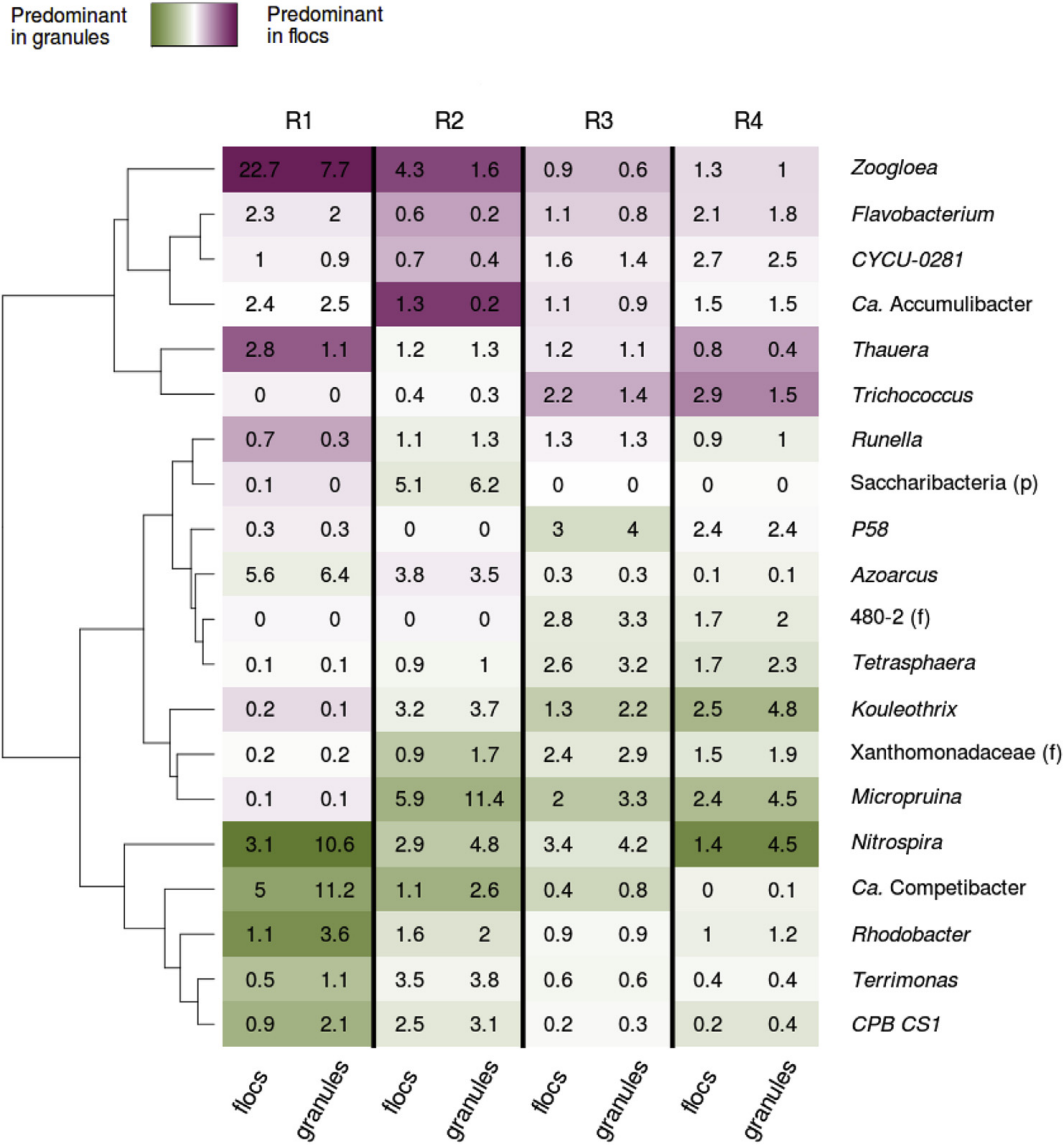
Average relative abundance of the main genera in the flocs and granules fractions collected in the four reactors during the stable state. Purple indicates a higher proportion in flocs while green indicates a higher proportion in granules. A pseudo-count of 0.5% was added to each abundance to lower the possible effect of the noise in very low abundant genera. (For interpretation of the references to color in this figure legend, the reader is referred to the Web version of this article.)

The presence of non-diffusible organic substrate in the WW resulted in 20–40% of flocs in the AGS. It is thus relevant to understand to what extent the microbial communities in flocs and granules are similar. The relative abundances of the main genera in flocs and granules were compared to detect potential enrichment of some genera between those two types of microbial aggregate (Fig. 6). At stable state, *Zoogloea*, *Flavobacterium*, *CYCU-0281*, *Thauera* and *Trichococcus* were enriched in flocs, whereas *Nitrospira*, Ca. *Competibacter*, *Rhodobacter*, *Terrimonas* and *CPB_CS1* were enriched in granules. However, only few of these differences were significant (Supplementary Information Table S10) when considered separately, *e.g. Nitrospira* in R1 and R4 and *Thauera* in R4. Hence, in term of microbial community composition, flocs are quite similar to granules.

### 3.5 Nutrient removal performance

Overall, good substrate/nutrient removal and effluent quality was observed for all systems, despite significant differences in the sludge properties and granulation process (Table 2). Very low TSS concentrations were measured in the effluent of R1 and R2 (<20 mgTSS L^−1^), as well as in the effluent of R4 run#1 (15 mgTSS L^−1^). The highest TSS concentration was measured in the effluent of R3 (45 mg TSS L^−1^). During run#2 of R4, TSS effluent concentrations (34 mgTSS L^−1^) were lower than in R3, but still higher in comparison to R4 run#1 as well as R1 and R2.

**Table 2:** Effluent concentrations and nutrient removal performances of the four AGS reactors fed with 100%-VFA synthetic (R1), complex synthetic (R2), primary effluent (R3) and raw influent (R4) WW run#1 and run#2 a^,^^b^.

	100%-VFA synthetic WW	Complex synthetic WW	primary effluent WW	raw WW	raw WW
	R1	R2	R3	R4 run#1	R4 run#2
TSS effluent [mgTSS L^−1^]^c^	13 ± 15	17 ± 22	43 ± 43	15 ± 15	34 ± 34
COD removal [%]	91 ± 7	93 ± 5	83 ± 11	92 ± 3	88 ± 8
TN removal [%]	77 ± 14	60 ± 16	45 ± 20	47 ± 12	63 ± 16
NH_4_–N removal [%]	95 ± 7	97 ± 6	96 ± 4	94 ± 7	97 ± 5
NH_4_–N effluent [mgN L^−1^]	0.2 ± 0.4	0.1 ± 0.1	0.3 ± 0.5	0.2 ± 0.2	0.2 ± 0.3
NO_3_–N effluent [mgN L^−1^]	4 ± 4	13 ± 5	13 ± 6	12 ± 4	11 ± 5
TP removal [%]	89 ± 10	89 ± 14	49 ± 44	64 ± 20	73 ± 17
PO_4_–P removal [%]	92 ± 11	96 ± 7	64 ± 28	63 ± 24	79 ± 18
PO_4_–P effluent [mgP L^−1^]	0.4 ± 0.6	0.2 ± 0.3	1.2 ± 1.1	1.0 ± 0.7	0.7 ± 0.9

a Averageand SD were calculated from 29 to 39 measurements for R1, R2, R3, 12–15 for R4 run#1, and 16–24 for R4 run#2, respectively.

b NO_2_–N in the effluent was in the range of 0.1–0.3 mgN L^−1^ for all reactors.

c Calculated from measurements of samples taken during stable operation (no sludge washout events).

Excellent COD- and NH_4_–N-removal efficiencies were observed in all reactors, except R3. For R1, R2, and R4, the COD- and NH_4_–N-removal efficiencies were consistently larger than 90% and 95%, respectively. The sludge loss in the effluent of R3 (43 ± 21 mgTSS L^−1^) increased the average COD concentration in its effluent. Effluent concentrations of NH_4_–N and NO_2_–N were consistently low in all reactors. High TN-removal (77%) *via* simultaneous nitrification-denitrification (SND) was observed in R1 only, which was indicated by the lowest NO_3_–N effluent concentrations. Larger NO_3_–N effluent concentrations measured in the effluent of R2, R3 and R4 resulted from a larger accumulation of NO_3_–N during the aerobic phase, compared to R1. Since full nitrification was observed in all systems, it can be concluded that the higher NO_3_–N effluent concentrations result from a lower (simultaneous) denitrification rate during aerobic bulk conditions.

PO_4_–P-removal was constant and high (>90%) for AGS of R1 and R2 only. AGS of R4 run#1 and run#2 showed lower PO_4_–P-removal of 61 and 78% on average, respectively. The lowest PO_4_–P-removal was observed for AGS of R3. However, PO_4_–P-removal of R3 and R4 run#2 improved up to >95% after introducing anaerobic PF + mixing at day 357 and 289, respectively.

Overall, successful start-up of the reactors in terms of substrate/nutrient removal was achieved long before achieving good settling properties. Full COD and NH_4_–N removal were observed right after inoculation of the systems. High P-removal was observed without delay for R1 and R2 while stable and high biological phosphorus removal was reached after 77 and 93 days for R3 and R4 run#2, respectively. R4 run#1 was not able to recover high P-removal performance before the restart.

### 3.6 Correlations between settling properties, nutrient-removal performances and microbial community composition

**Fig. 7:**
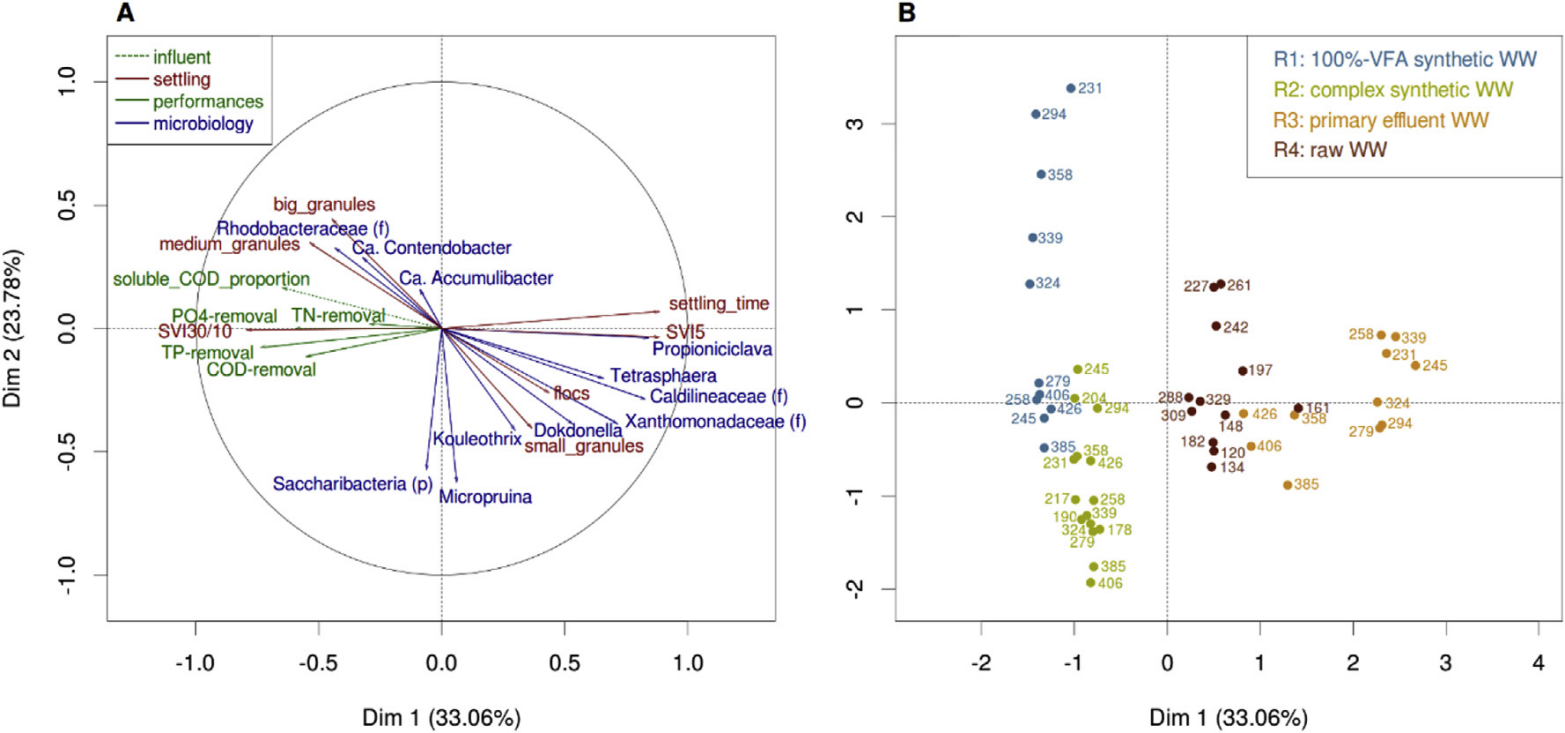
Multiple factor analysis (MFA) performed on the data at bacterial stable state in the four reactors with three groups of variables: settling characteristics, nutrient-removal and composition of the bacterial communities. These graphs show the contribution of the settling characteristics the nutrient-removal and the bacterial community compositions to the two first axis (A), and the projection of the corresponding sample points in this two-dimensional space (B).

Multiple factor analysis (MFA) indicates a correlation between the proportion of soluble COD in the influent, a high proportion of medium to big granules and a SVI_30/10_ ratio close to 1, suggesting good granulation and settleability (Fig. 7). The proportion of soluble influent COD also correlated with good nutrient-removal performances, such as nitrogen, total phosphorus or phosphate removal efficiencies (TN, TP and PO_4_–P-removal). The projection of the samples in the two-dimensional MFA space provides information about the global similarity between samples (Fig. 7B). Overall, samples of R2 are close to the ones of R1 and closer to samples of R4 than R3. At stable state, the bacterial communities, settling properties, size distribution of the sludge, and the nutrient removal performances show different individual distributions in the two-dimensional space (Supplementary Information Fig. S17). Bacterial communities of AGS of R3 and R4 are very similar at stable state, and close to the communities of the inoculum (Supplementary Information Fig. S17A). Microbial communities of R3 and R4 are clearly distinct from the ones of the reactors treating synthetic WW, however closer to the ones of R2 than to those of R1. The projection of the samples forms a gradient from R1 to R3, with R2 and R4 in between, based on settling properties and size distribution of the sludge (Supplementary Information Fig. S17B). The links between the microbial communities and the size and density of the biomass is confirmed by the correlation of 0.65 between the two corresponding Bray-Curtis distance matrices (Supplementary Information Table S13 and Table S12). Based on the nutrient-removal performance data, a majority of samples mainly from R1, R2 and R4 grouped together. Outliers, mainly from R3, surrounded this group (Supplementary Information Fig. S17C). This reflects the fact that, AGS of R3 had lower nutrient-removal performances than AGS of the other three reactors, even at stable state. Weak correlation (0.05) between the Bray-Curtis distance matrices of the bacterial communities and the nutrient-removal performances (Supplementary Information Fig. S17) is observed at stable state.

### 3.7 Identification of correlations between the discriminant taxa and the sludge size distributions, the settling properties, and the nutrient-removal performances

**Fig. 8:**
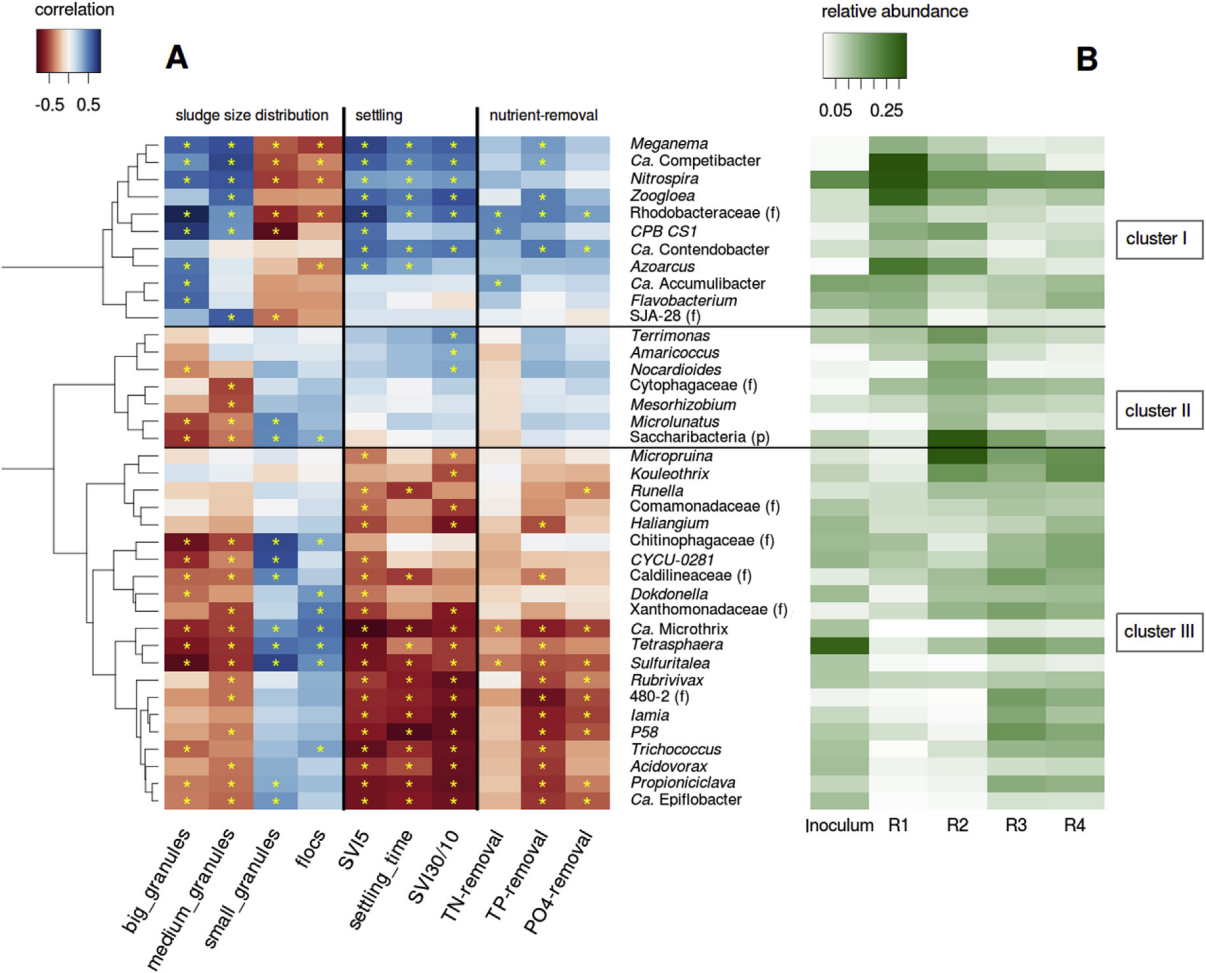
Correlation heatmap between the discriminant taxa and *Ca*. Accumulibacter and the sludge size distribution, the settling properties and the nutrient-removal efficiencies of the samples collected during the stable state, in the four reactors (A). The correlations having p-values lower than 0.01 are indicated with a yellow star. The different taxa were clustered together according to the similarity in terms of correlations with the different parameters of the sludge. The inverse values of SVI_5_ and settling time were used for the construction of the correlation heatmap. The average relative abundance of the taxa (Supplementary Information S14) after Hellinger transformation, in the four reactors during the stable state is indicated in green (B). (For interpretation of the references to color in this figure legend, the reader is referred to the Web version of this article.)

Three different clusters were identified by correlating the discriminant taxa with the different properties of the sludge (Fig. 8). Cluster I correlated with a high proportion of big and medium size granules, good settling properties and good nutrient-removal. It includes the PAO *Ca*. Accumulibacter and its GAO competitors from the family Competibacteraceae (*Ca*. Competibacter, *Ca*. Contendobacter, *CPB_CS1*). Aerobic filamentous (*Meganema*, *Zoogloea*), potentially filamentous (*Flavobacterium*) bacteria and the nitrifying *Nitrospira* also belong to this cluster. Numerous putative denitrifiers are also part of cluster I (*Zoogloea*, *Ca.* Competibacter, *Ca.* Contendobacter, *Ca*. Accumulibacter).

Cluster II correlates with high proportions of small granules and flocs, relatively good settling properties, good P-removal and partial TN-removal. It is composed of the aerobic bacteria *Terrimonas,* which has the ability to hydrolyse various substrates, and *Amaricoccus,* which can store carbon in the form of PHA. It also comprises fermentative or putatively fermentative bacteria such as *Microlunatus* and *Mesorhizobium* for which *in situ* physiology is not well described yet. The bacteria belonging to this cluster were relatively abundant in the reactor treating complex synthetic WW (R2).

Cluster III contains taxa correlated with high proportions of small granules and flocs, poorer settling properties, and lower nutrient-removal. This cluster contains one third of novel or poorly characterised genera such as *P58* or *Dokdonella*. It also includes fermentative bacteria including *Tetrasphaera*, *Micropruina*, *Propioniciclava Kouleotrix,* and *Trichococcus*. As cluster I, it contains various potential denitrifiers such as *Iamia*, *Sulfuritalea*, *Microthrix* or *Acidovorax*. Cluster III also comprises bacteria likely able to degrade macromolecules, *e.g. Ca*. Epiflobacter, *CYCU-0281* and members of the family Chitinophagaceae. These bacteria are more abundant in the systems treating municipal WW (Inocula, R3, R4). Members of the genera *Ca*. Microthrix and *Trichococcus,* and the family Caldilineaceaepresent in cluster III can be filamentous and thus impair the settleability of the sludge and be at least in part responsible for the poorer settleability associated with this cluster.

## 4 Discussion

### 4.1 Diffusibility of organic substrates has significant influence on formation of AGS

Wastewater composition in terms of diffusible/non-diffusible organic substrate significantly influences the formation of AGS. A comparison between the four reactors fed with different amounts of diffusible/non-diffusible organic substrate helped validating the conceptual model presented above (Fig. 1). Fast granulation was observed with WWs containing high amounts of diffusible organic substrate (R1 and R2), thus resulting in excellent settling properties and stable nutrient-removal performances. On the other hand, a low amount of diffusible organic substrate resulted in slow granulation, poorer settling properties, and often partial nutrient removal.

Granulation results from the selection of slower growing carbon-storing microorganisms over OHO (de Kreuk and VAN Loosdrecht, 2004; Vjayan and Vadivelu, 2017). The high proportions of diffusible organic substrate in R1 and R2 promoted the growth of organisms that can store or use carbon under anaerobic conditions. Storing microorganisms such as *Ca*. Accumulibacter (Classical PAO) and *Ca*. Competibacter or *CPB C22&F32* (classical GAOs) represented an important part of the microbial community of R1 (Fig. 4). In R2, the fermentative PAO *Tetrasphaera* and the fermentative GAO *Micropruina* were abundant, in particular during granulation. The influent composition specifically favoured the growth of these organisms, which coincided with the rapid development of well-settling AGS. However, the size of the granules which developed in R1 and R2 was very different, with large granules dominating AGS of R1 while smaller granules were observed in R2. If diffusibility of substrate is very high, bacterial growth in the deep layers is then promoted, ultimately leading to the formation of dense/large granules as in R1. Granule size is in theory linked to deep substrate penetration into the granule, which is influenced by the substrate concentration gradient, uptake rate, and diffusibility of substrate (Morgenroth, 2008; Rittman and Mccarty, 1981). The nature and content of extracellular polymeric substances (EPS) – associated with granule densification - is influenced by substrate type and loading, and has strong implications on the granule size and settling (Rusanowska et al., 2019). Deep substrate diffusion and conversion occurred in R1 as indicated by the high SND measured for this system. During the aeration phase, denitrification occurred in the anoxic zones of the granules due to previous anaerobic storage of diffusible organic substrate. Classical PAO and GAO enriched in the AGS of R1 were able to grow under both anoxic and aerobic conditions thus favouring the densification of the granules. The lower fractions of diffusible organic substrate in R2 influent resulted in a limited growth in the core of the granules and ultimately in the formation of smaller granules. The different properties of the granules of R1 and R2 (settling, granule size and biomass fractionation, etc.) can thus be explained by the amount and nature of the diffusible and non-diffusible substrates (VFA, fermentable soluble and particulate organic substrate). Granulation can therefore be linked to two aspects: (1) the diffusibility of the substrate governs its availability within the granules and (2) the nature of the substrates determines the microbial community composition and the aggregates densification.

On the contrary, the high proportions of non-diffusible X_B_ in the influent of R3 and R4 hampered the granulation process. Several phenomena lead to the conclusion that hydrolysis is only partial during anaerobic plug-flow feeding: (1) WW particles are often large, from several μm up to 1–2 mm (Levine et al., 1985; Dimock and Morgenroth, 2006), (2) hydrolysis is a very slow process (Benneouala et al., 2017; Morgenroth et al., 2002; Jabari et al., 2016), and (3) the anaerobic feeding duration of 1–2 h typically applied in AGS systems is insufficient to provide full hydrolysis of X_B_ (Wagner et al., 2015; Jabari et al., 2016). Therefore partial anaerobic conversion of X_B_ is unavoidable and results in a high availability of substrate during the subsequent aerobic phase, which in turn supports the growth of OHO (Wagner et al., 2015). OHO growth leads to poorer settling properties due to filamentous outgrowth, floc formation or a decrease of P-removal (Novák et al., 1993; Suresh et al., 2018; Weissbrodt et al., 2014; Pronk et al., 2015a; de Kreuk et al., 2010). Extensive OHO growth outcompeting slower growing storing organisms is the most likely reason for the slow development of granules, bad settling properties and overall lower nutrient removal performance observed for AGS of R3 and R4, in comparison to R1 and R2. Classical PAO and GAO were outcompeted by fermentative PAO and GAO in R3 and R4, and similarly in R2. The enrichment of fermentative PAO and GAO in those systems is the result of lower influent VFA concentrations combined with large amounts of fermentable substrates, stemming from both influent WWs and produced *via* hydrolysis of non-diffusible compounds.

### 4.2 Start-up

High concentrations of diffusible organic substrate, as found in the synthetic WWs of R1 and R2, led to faster granulation (1 month) compared to granulation in R3 (>1 year) or R4 (5 months). A main finding of our study is that different characteristic times were required to establish stable microbial communities, stable physical properties of the AGS, or steady substrate/nutrient removal. Characteristic time to establish full substrate/nutrient removal was the quickest (around few days/weeks) while characteristic time to establish a stable microbial community was the slowest (several months).

Complete substrate and nutrient removal was observed without any delay right after inoculation with activated sludge for all reactors. Maintaining high conversion rates after inoculation required an appropriate start-up strategy. Our selected start-up strategy relied on applying a low washout stress to avoid a too harsh washout of slow growing organisms. Full nitrification without NO_2_^−^ accumulation was observed after start-up, while no or a very short loss of biological P-removal was noticed. Sustained nutrient removal after inoculation was also observed by Lochmatter and Holliger (2014), who applied a similar start-up strategy of low washout stress on slow-settling biomass. Low amounts of diffusible organic substrate in the WW must thus be balanced by a less harsh washout of flocs during start-up.

Aggressive washout of slow settling biomass as strategy to start-up AGS systems using either 100%-VFA or municipal WW as influent can result in very short start-up times down to 1 and 3 weeks, respectively, with settleability of SVI_10_ < 40 mL g^−1^ (de Kreuk and VAN Loosdrecht, 2004; de Kreuk and VAN Loosdrecht, 2006). The current study did not apply aggressive washout of slow settling biomass (flocs) during start-up resulting in significantly slower granule formation. Since granulation was achieved in all reactors, the start-up strategy in the current study is viable and relevant even in unfavourable influent WW conditions, such as low strength municipal WW. Maintaining high substrate/nutrient conversion rates over long-term ultimately resulted in the formation of granules. The different kinetics of AGS formation confirm the general trend observed in previous studies. Start-up is typically shorter with diffusible-only influent WW, consisting of 100%-VFA (1–4 weeks) (de Kreuk and VAN Loosdrecht, 2004; Lochmatter and Holliger, 2014; Weissbrodt et al., 2014), than with WW containing non-diffusible polymeric compounds, such as municipal or industrial WW (3 weeks – more than 1 year) (de Kreuk and VAN Loosdrecht, 2006; Liu et al., 2010; Giesen et al., 2013). The faster granulation of AGS in R4 compared to AGS of R3 confirms that higher organic loads facilitate the granulation process (Nancharaiah and Kiran Kumar Reddy, 2017; Li et al., 2008; Rusanowska et al., 2019).

Establishing a stable microbial community required between 4 and 8 months for all systems. During this period, different transient bacterial communities were observed. The transient bacterial community was dominated by *Zoogloea* in R1, fed with 100%-VFA WW. We propose that the presence of *Zoogloea* in R1 is an indirect consequence of the excellent settling properties of the AGS, which led to preferential flow and bypass of soluble COD into the aerobic phase (Supplementary Information S15). The growth of *Zoogloea* during the early stage of granulation in R1 likely resulted from the presence of VFA in the aerobic phase (Weissbrodt et al., 2013). Similar to the study of Weissbrodt et al. (2013), the high abundance of *Zoogloea* observed in R1 was associated with a high proportion of granules and a thin settled bed during feeding. Several studies however suggested that *Zoogloea* plays a positive role in the formation of granules in VFA-rich influent WW by producing specific EPS (Li et al., 2008; Larsen et al., 2008; Kang et al., 2018). Therefore, the availability of diffusible substrates under aerobic conditions might not automatically be detrimental to granulation. In the reactors fed with complex WW (R2, R3 and R4), Actinobacteria dominated the transitional bacterial community. In particular, *Micropruina* was very abundant in these three reactors and was concomitant to the formation of granules. Its role in granulation in complex WW fed AGS systems is yet to be determined.

### 4.3 Stable state

The list of potential organisms that play a functional role in AGS systems can be extended based on this study considering their effect on nutrient removal, and taking into account the role of different influent compositions. However, a core microbial community of AGS systems cannot be established yet. During stable state operation, dominant species in R1 were reported as abundant in other 100% VFA-fed AGS systems, such as *Zoogloea*, *Thauera*, *Rhodobacter*, *Meganema* and *Nitrospira*. In systems fed with VFAs only, the PAO guild mainly consisted of *Ca*. Accumulibacter while the GAO guild was mostly composed of members of the Competibacteraceae family (*e.g.*, *Ca*. Competibacter, CPB_C22&F32) (Weissbrodt et al., 2013; He et al., 2016a; Henriet et al., 2016). Such “simple” microbial community greatly differs from the ones of R2, R3 and R4. Abundant taxa detected in R2, R3 and R4 were previously detected in AGS fed with complex WW, like, *e.g.*, *CYCU-0281*, *Dokdonella*, *Flavobacterium*, *Haliangium*, *Nitrospira*, *Rhodobacter*, *Thauera*, *Thrichococcus*, unclassified genera related to *Xanthomonadaceae,* and *Zoogloea* (Kang et al., 2018; Szabó et al., 2017a; Świątczak and Cydzik-Kwiatkowska, 2018). Fermentative bacteria were present in both R2 and R3/R4, in particular the PAO *Tetrasphaera* and the GAO *Micropruina*. The sole presence of both diffusible and non-diffusible organic substrate – independent of their nature - resulted in relatively similar microbial communities. Yet, the remaining differences between those systems could stem from continuous inoculation of the sludge by bacteria present in the influent WW. Indeed, the similarity of the bacterial communities of R3 and R4 is stronger within AGS sampled during the same date than after the same number of days of reactor operation. Parts of the microbial communities thus stem from immigration of bacteria *via* the influent WW (Saunders et al., 2016). Filamentous OHO detected in high proportions in R3 and R4 (*e.g.*, *Trichococcus* and the family Caldilineaceae) can have a negative impact on the settling properties of the sludge. Those filamentous OHO are characterised by a high affinity for aerobic carbon degradation, which is consistent with the presence of slowly biodegradable substrates in the influent of those reactors. Yet, many genera known to have hydrolysing capabilities, such as *Ca*. Epiflobacter, *CYCU-0281* and *Kouleothrix,* correlated with low settling properties because they are linked to the presence of X_B_, but not necessarily because they are filamentous.

During stable state, the bacterial communities selected in the different AGS systems had multiple taxa in common with the core communities of EBPR activated sludge (*e.g.*, *Ca*. Accumulibacter, *Micropruina*, *Tetrasphaera*, *Zoogloea*) (Saunders et al., 2016; Stokholm-Bjerregaard et al., 2017). But many taxa that are not yet characterised at the genus level were identified. They belong to the family of Xanthomonadaceae, Caldilineaceae, Cytophagaceae or to the phylum of Saccharibacteria. However, their function in AGS systems is yet to be determined.

The grouping of R1 vs. R2/R3/R4 based on microbial community also reflected differences in TN-removal, which was lower in the reactors fed with complex WW. TN-removal in the systems can occur either *via* (1) pre-denitrification of remaining NO_3_^−^ from the previous cycle during the feeding and subsequent anoxic/anaerobic mixing phase, or (2) SND during aerobic bulk conditions. The latter process requires denitrifying bacteria, available COD and the presence of substantial anoxic zones within the granules. Numerous putative denitrifying bacteria were detected in AGS of all reactors, but only R1 gathered all the conditions to perform SND resulting in low effluent NO_3_^−^; large granule size, and a high proportion of diffusible organic substrate in the influent. Further research is required to identify the influence of each of these factors on SND, and to improve SND in AGS systems fed by complex influent WW. Decreased P-removal was observed in AGS systems treating municipal WW (R3, R4), possibly due to low influent diffusible organic substrate (especially VFA) in combination with carbon leakage, and low influent PO_4_–P concentrations (Guimarães et al., 2018). Short-term loss of P-removal was also observed in R1 concomitantly to bypassing of substrate during PF feeding. In addition, low (ortho-) P concentrations of the municipal WW received by R3/R4 did not promote the growth of PAOs (de Kreuk et al., 2010). Indeed, the proportion of PAOs was above 5% in the four reactors at stable state. These proportions are much lower than the average proportion reported in Danish EBPR WWTP (13%) and may have made the four systems less robust in terms of P-removal (Nielsen et al., 2010). Yet, the anaerobic carbon uptake, and therewith P-removal, were enhanced by the introduction of an anoxic/anaerobic mixed phase after PF feeding.

### 4.4 The role of flocs in AGS systems

Our results indicate that the presence of flocs (20–40%) is representative of AGS systems fed with WWs that contain non-diffusible X_B_ (Fig. 3). Flocs were observed several months after the establishment of good/stable settling properties and substrate/nutrient removal. Flocs fractions ranging from 16 to 40% of TSS were also reported in literature for pilot- and full-scale AGS plants (Derlon et al., 2016; Pronk et al., 2015b; van Dijk et al., 2018). Our results thus indicate that AGS systems are hybrid systems, composed of both flocs and granules, rather than biofilm (only) systems.

The presence of flocs in AGS systems results from both short-term and long-term mechanisms. Short-term exposure of the granules to non-diffusible X_B_ triggers filamentous outgrowth (de Kreuk et al., 2010). X_B_ attaches to the granule surface and is then partially hydrolysed during the anaerobic phase (de Kreuk et al., 2010). The fraction of X_B_ that is not converted anaerobically is then degraded under aerobic conditions, thus promoting the growth of OHO and filamentous outgrowth (finger-type) (Pronk et al., 2015a). Such short-term mechanism might however not be relevant for real AGS systems, in which the sludge is exposed to X_B_ over long-term (several months/years). Over long-term, the presence of X_B_ in the influent favours the presence of flocs. Filamentous structures in turn do not develop on the surface of granules as X_B_ is likely captured/degraded by flocs (Derlon et al., 2016; Wagner et al., 2015). X_B_ attachment onto the granules surface is likely limited during anaerobic PF-feeding, while the hydrolysis rate is in addition very low, especially under anaerobic conditions (Jabari et al., 2016). For these reasons, it is expected that significant amount of X_B_ remain available during the mixed aerobic phase. We hypothesized that under mixed conditions, flocs would have a competitive advantage over granules for capturing and then degrading X_B_ (Wagner et al., 2015; Derlon et al., 2016). The surface-to-volume ratio of the flocs is much larger than the one of the regular and round shaped granules (Andreadakis, 1993; Mihciokur and Oguz, 2016). We propose that the availability of X_B_ during the mixed aerobic phase combined with its selective capture by flocs is the main reason for their presence in AGS systems treating municipal WW. We ultimately suggest that flocs might in fact be beneficial during treatment of complex WW with elevated levels of non-diffusible X_B_ using AGS technology.

The microbial communities in the flocs and granules differed only for some particular genera at stable state. Thus the microbial community structures evolved in a similar manner in both fractions, probably due to a constant exchange of biomass between the two fractions (Liu et al., 2010; Zhou et al., 2014). For the four reactors, our results indicate a higher fraction of *Zoogloea* in flocs than in granules. The enrichment of *Zoogloea* in flocs was particularly pronounced in the AGS of R1, where the proportion of flocs was the lowest (5%) and where filamentous outgrowth was often observed. The presence of *Zoogloea* in flocs likely resulted from erosion of the granules' surface (Szabó et al., 2017b). Erosion of the granule's surfaces might have been favoured by the low cohesion of filamentous structures resulting from the growth of *Zoogloea*. On the contrary, the higher abundance of slow growing organisms in granules, *e.g.*, *Nitrospira,* likely resulted from favourable growth conditions in the core of the granule. The SRT gradually increases over the granule's depth, thus providing suitable growth conditions for slow growing organisms such as nitrifiers. High cohesion within granules also reduces the detachment rate and thus the exchange of bacteria from the granules to the flocs. In this case, the differences between the bacterial communities of granules and flocs can become significant, because both fractions offer different niches and have different retention times (Winkler et al., 2012). It is therefore expected that slow growing bacteria are progressively enriched in the granules. The mechanism is confirmed by our measurements of relative abundance of *Nitrospira*, *Ca*. Competibacter or *CPB C22&F32*. Moreover, the lowest differences between the microbial communities of flocs and granules in R3 can be explained by granulation being more recent compared to the other systems.

### 4.5 Implications for research and practice

Our findings have relevant implications for both research and engineering practice. The complex synthetic WW resulted in the development of AGS that was more similar to AGS fed by municipal WW rather than AGS fed by100%-VFA. We therefore advise the use of complex synthetic WW (VFA, diffusible fermentable substrate and high non-diffusible X_B_ contents) as surrogate of municipal WW. The proportion and composition of X_B_ can be tuned and modified to address specific research questions.

In terms of implications for engineering practice, our study provides relevant information for the start-up of AGS systems. The superior performance of AGS in R4 over AGS in R3 indicates that an increased loading was beneficial for granulation and nutrient-removal, despite the increased fraction of X_B_ in the influent. However, a balance must be found between applying a sufficient loading while limiting operating costs (*e.g.*, aeration). Start-up time was likely extended due to the chosen start-up strategy (low washout stress and low v_ww_ during PF feeding) in comparison to other studies (Derlon et al., 2016; de Kreuk and VAN Loosdrecht, 2006). But operating the system at low v_WW_ also helped maintaining high substrate and nutrient removal rates during the entire experimental phase, as observed in the present study and reported in literature (Derlon et al., 2016; Lochmatter and Holliger, 2014). Applying a higher selective pressure by gradually increasing the v_ww_ during PF feeding would accelerate the formation of granules. But based on our experience, increasing the selective pressure applied *via* v_crit_ is coupled with an increased risk of biomass loss. Due to the sensitivity of AGS systems fed by low-strength municipal WW, a fine balance between maintaining high SRT conditions for forming granules and applying a selective sludge removal of slow settling biomass must be found.

In terms of nutrient removal, partial denitrification due to poor SND proved to be representative of AGS systems fed with non-diffusible X_B_. An increased TN-removal in those systems was achieved by implementing an additional mixed phase following the PF feeding. Denitrification during this phase likely benefited PO_4_–P removal, as observed for R3 and R4.

## 5 Conclusions

The main conclusions of this study are:

1. The wastewater composition in terms of diffusible and non-diffusible organic substrates governs both the microbial community composition, granulation kinetics, settling properties, and nutrient removal of AGS. High fractions of diffusible organic substrates result in fast granulation and excellent settleability of AGS, whereas presence of non-diffusible X_B_ in the influent hampers granulation, reduces settleability, and results in the presence of substantial fractions (20–40%) of flocs. The bacterial communities of flocs and granules were globally very similar within the same reactor, but several taxa were enriched in flocs or granules, respectively.
2. AGS fed by VFA based synthetic WW resulted – as expected - in a specialized bacterial community containing classical PAO and GAO (*e.g.*, *Ca.* Accumulibacter, *Ca.* Competibacter or *CPB C22&F32*) that led to fast granulation, excellent settling performance, stable nutrient removal, large granules, and a quasi-absence of flocs.
3. AGS fed by complex substrates, containing non-diffusible X_B_, revealed bacterial communities characterised by a high abundance of fermenting bacteria, including fermentative PAO and GAO. High amounts of diffusible organic substrate and total organic load were key factors to enhance the settleability and granulation kinetics of the sludge as well as the stability of nutrient-removal performances.
4. An increased floc fraction was constitutive in AGS reactors fed with complex WW and was attributed to the presence of non-diffusible X_B_ in the influent. It is neither possible nor desirable to wash out all flocs in AGS systems fed with complex WW.
5. Complex synthetic WW led to AGS with characteristics resembling those treating raw municipal WW. Hence, the often applied 100%-VFA synthetic WW should be replaced by complex synthetic WW as a surrogate of municipal WW in future lab-scale experiments studying AGS.

## Declaration of interests

The authors declare that they have no known competing financial interests or personal relationships that could have appeared to influence the work reported in this paper.

## Appendix A Supplementary data

Supplementary Information Supplementary Information

S9_discriminant_and_p_value_t_tests.xlsx S9_discriminant_and_p_value_t_tests.xlsx

S12_bray_distance_matrices_stable_state.xlsx S12_bray_distance_matrices_stable_state.xlsx

S14_genera_mean_abundance_at_stable_state.xlsx S14_genera_mean_abundance_at_stable_state.xlsx
