# Supplementary material for: Organic substrate diffusibility governs microbial community composition, nutrient removal performance and kinetics of granulation of aerobic granular sludge
Source: Water Res X. 2019 May 20;4:100033. doi: 10.1016/j.wroa.2019.100033 (PMC6614711; doi:10.1016/j.wroa.2019.100033)
Supplement: Supplementary Information [file mmc1.pdf]

Organic substrate diffusibility governs microbial community composition, nutrient removal performance and kinetics of granulation of aerobic granular sludge

SUPPLEMENTARY INFORMATION

M. Layer[*,**], A. Adler[***], E. Reynaert[*,***], A. Hernandez[*,***], M. Pagni[****], E. Morgenroth[*,**], C. Holliger[***], N. Derlon[*]

* Eawag, Swiss Federal Institute of Aquatic Science and Technology, Überlandstrasse 133, 8600 Dübendorf, Switzerland

** ETH Zürich, Institute of Environmental Engineering, 8093 Zürich, Switzerland

*** Ecole Polytechnique Fédérale de Lausanne (EPFL), ENAC IIE Laboratory for Environmental Biotechnology, 1015 Lausanne, Switzerland

**** SIB Swiss Institute of Bioinformatics, 1015 Lausanne, Switzerland

Emails of the authors: Manuel.Layer@eawag.ch, Aline.Adler@epfl.ch

Corresponding author: Nicolas Derlon: Nicolas.Derlon@eawag.ch

Table S1: Composition of trace element solution, after preparation pH is adapted to 6 using KOH (30% v/v).

**Component**	**Formula**	**Concentration [g L^{-1}]**
EDTA disodium salt dihydrate	$C_{10}H_{14}N_2Na_2O_8$ * $2H_2O$	16.215
Zinc II Sulfate	$ZnSO_4$* $7H_2O$	0.44
Manganese II Chloride	$MnCl_2$* $6H_2O$	1.012
Ammonium Iron II	$(NH_4)_2Fe(SO_4)_2$* $6H_2O$	7.049
Ammonium Molybdate	$(NH_4)_6Mo_7O_{24}$* $4H_2O$	0.328
Copper II Sulfate	$CuSO_4$* $5H_2O$	0.314
Cobalt II Chloride	$CoCl_2$* $6H_2O$	0.322

Table S2: Recipe of influent WW of 100%-VFA synthetic WW and Complex synthetic WW receiving reactors R1 and R2. The recipes only provide C and N species to the wastewater preparation. The recipes were prepared in 20-fold concentration, to provide total COD and TN concentrations of 600 mg COD L^{-1} and 52 mg TN L^{-1}, respectively.

Component	100%-VFA synthetic WW<br>Concentration [g L^{-1}]	Complex synthetic WW<br>Concentration [g L^{-1}]
NaAcetate*$3H_2O$	12.8	4.3
NaPropionate	4.8	1.6
$(NH_4)Cl$	3.2	1.1
$CaCl_2$*$1H_2O$	0.35	0.35
$MgSO_4$	0.33	0.33
KCl	0.66	0.66
Glucose/Dextrose	-	1.9
Starch	-	1.4
Peptone	-	1.6
Alanine	-	0.27
Arginine	-	0.26
Aspartic acid	-	0.40
Glutamic acid	-	0.29
Glycine	-	0.45
Leucine	-	0.16
Proline	-	0.19

Protocol S3: Amplification of 16S rRNA gene by polymerase chain reaction.

The bacterial 16S rRNA gene hypervariable regions V1-V2 were amplified by PCR using the universal primers 27F and 338R (bold), respectively, (bold) with overhang adapters attached: forward (5’ TCGTCGGCAGCGTCAGATGTGTATAAGAGACAG-**AGMGTTYGATYMTGGCTCAG**3') and reverse (5’GTCTCGTGGGCTCGGAGATGTGTATAAGAGACA-**GGCTGCCTCCCGTAGGAGT**3'), and the High-Fidelity Q5 polymerase (High-fidelity 2x Master Mix, Biolabs Inc., USA). For each sample, 25 ng of DNA were mixed with forward primers 27F and reverse primers 338R for a final concentration of 0.5 uM each and completed with Q5 High-Fidelity 2x master mix water, for a final volume of 50ul. The PCR runs were performed in a T3000 Thermocycler (Biometra GmbH, Germany) with the following steps: initiation (2 minutes, 95°C), followed by 30 cycles of denaturation (45 seconds, 95°C), annealing (45 seconds, 50°C) and elongation (60 seconds, 72°C) and a final extension step (5 minutes, 72°C).

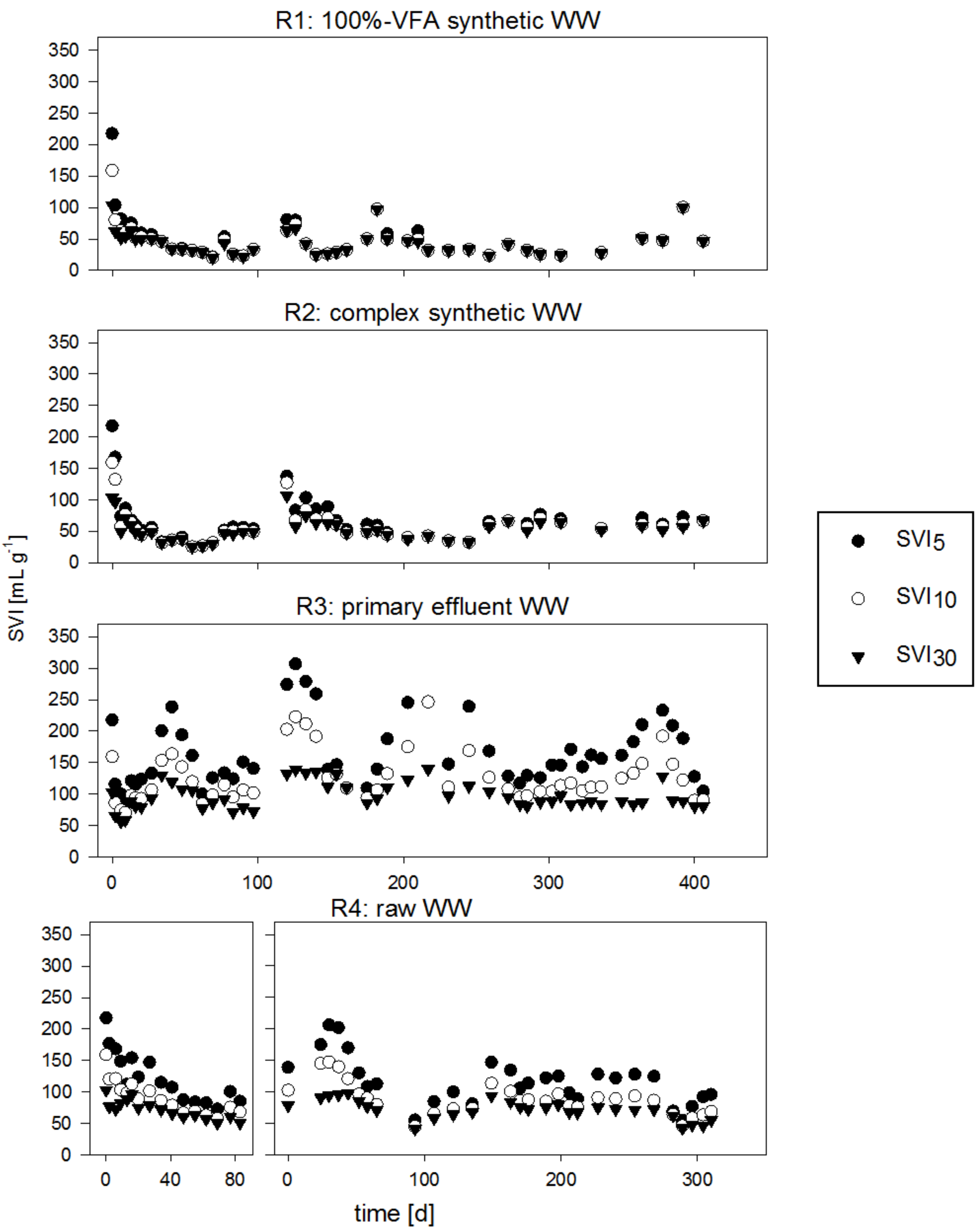

Figure S4: Evolution of SVI_5, SVI_{10} and SVI_{30} of R1, R2, R3, R4 run#1 and run#2

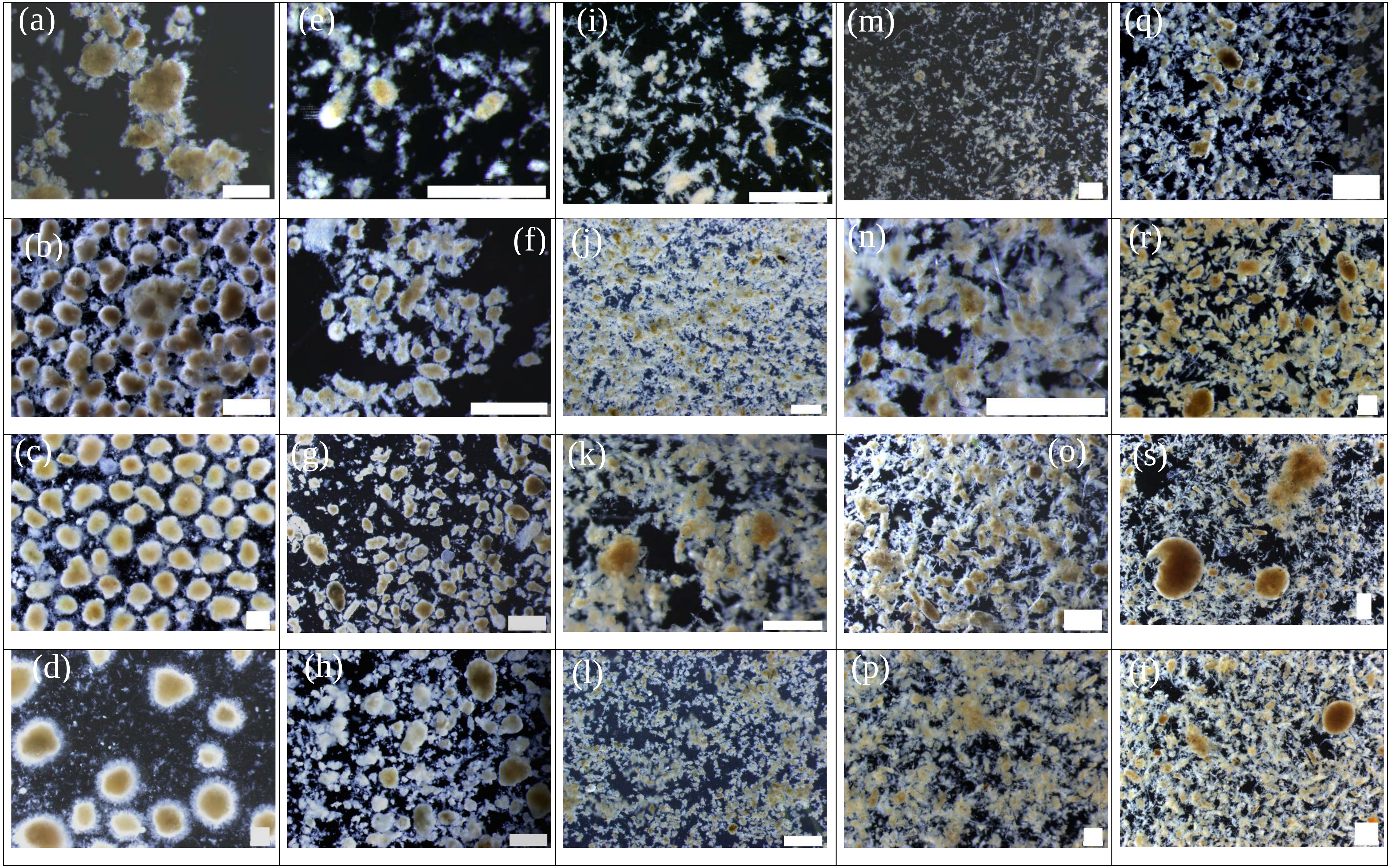
(a)
(e)
(i)
(m)
(q)
(b)
(f)
(j)
(n)
(r)
(c)
(g)
(k)
(o)
(s)
(d)
(h)
(l)
(p)
(t)

Figure S5: Evolution of sludge morphology in R1 fed by 100%-VFA synthetic WW (**a, b, c, d**) after 12, 57, 93, and 190 days of operation, in R2 fed with complex synthetic WW (**e, f, g, h**) after 12, 34, 57, and 100 days, in R3 fed with primary effluent WW (**i, j, k, l**) after 7, 147, 279, and 400 days, and in R4 fed with raw WW (**m, n, o, p** for run#1, **q, r, s, t** for run#2) after 7, 22, 57, and 84 days for run#1, and after 4, 100, 163, and 212 days for run#2. Size bars = 1.0 mm. Morphology of AGS fed by 100%-VFA influent WW (R1) significantly differed from the morphology of the AGS fed by complex synthetic WW (R2) and municipal WW (R3, R4). AGS of R1 was composed of large, round-shaped, dense and overall homogenous shaped aggregates that dominated overall sludge morphology (Figure S5**a-d**). Almost no flocs were observed. Operational issues of carbon leakage (Supplementary information Figure S15) from anaerobic to aerobic phase were the cause of filamentous outgrowth at the granules surface, visible on Figure S5**d**, after 190 days of operation. Visual observations indicated that AGS fed with complex influent WW (R2, R3, R4) were composed of both flocs and small and dense aggregates. Formation of “finger-type” granules was not observed in R2, despite the feed of particulate substances (starch). The highest complexity WW fed systems R3 and R4 also resulted in the most complex sludge morphology. In these reactors, aggregates with dense cores formed, but with very heterogeneous sizes and shapes. The formation of “finger-type” granules was observed (Figure S5**p** and **t**) but never over prolonged time. Large amounts of fibers or debris from the influent also accumulated in those systems (Figure S5**n** and **q**). First appearance of granules took much longer in complex WW fed reactors R2, R3 and R4 compared to R1. In R1 first granules were observed already after 12 days of operation (Figure S5**a**). The formation of granules in the complex WW fed reactors R2, R3 and R4 was much slower in comparison. In R2 it took 30 days until first round-shaped and dense granules were observed (Figure S5**f**). Granules only appeared after 100 days of operation in R4 run#2 and 279 days in R3. No large granules were observed in R4 during run#1, which was shorter than 100 days.

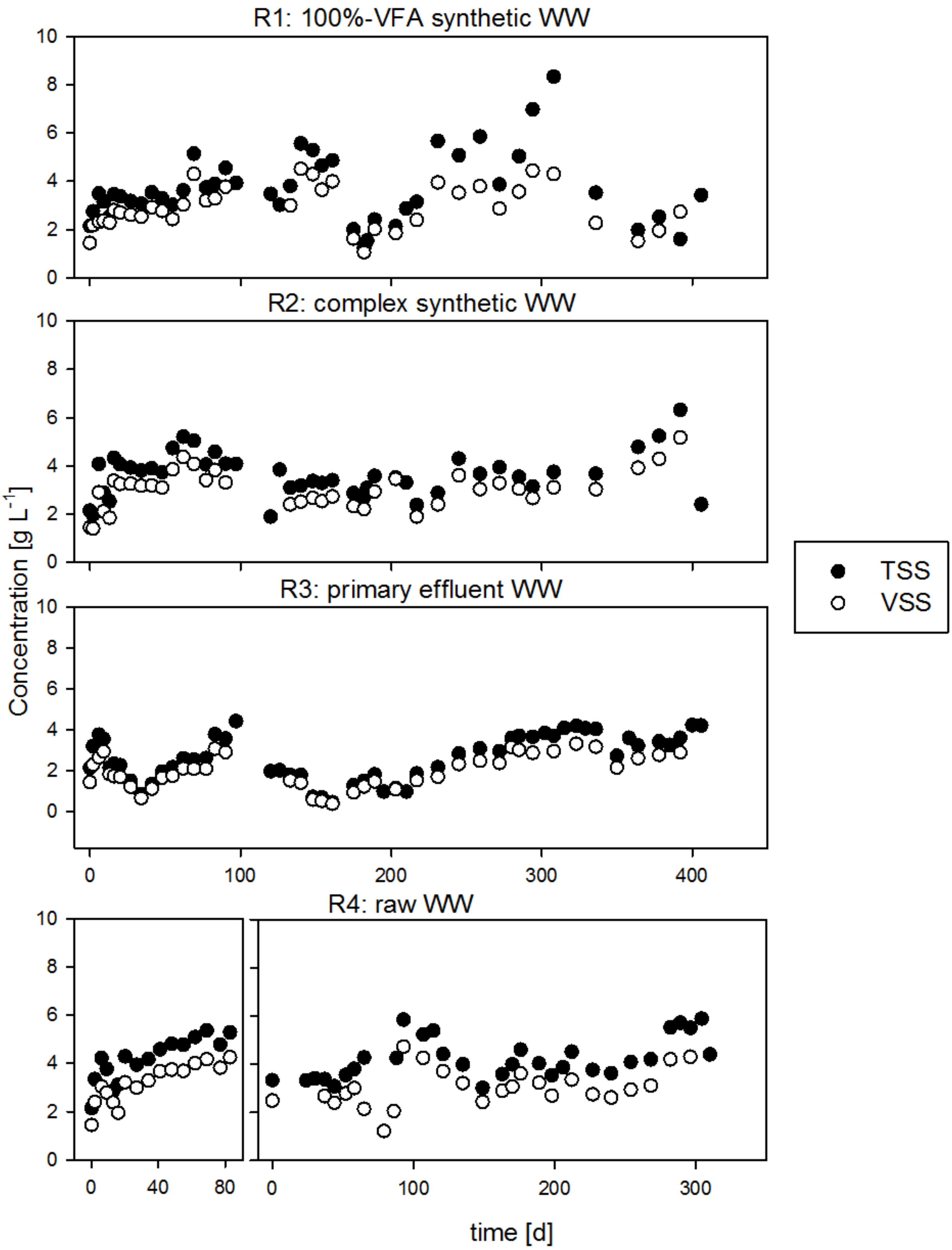

Figure S6: Evolution of TSS and VSS of R1, R2, R3, R4 run#1 and run#2

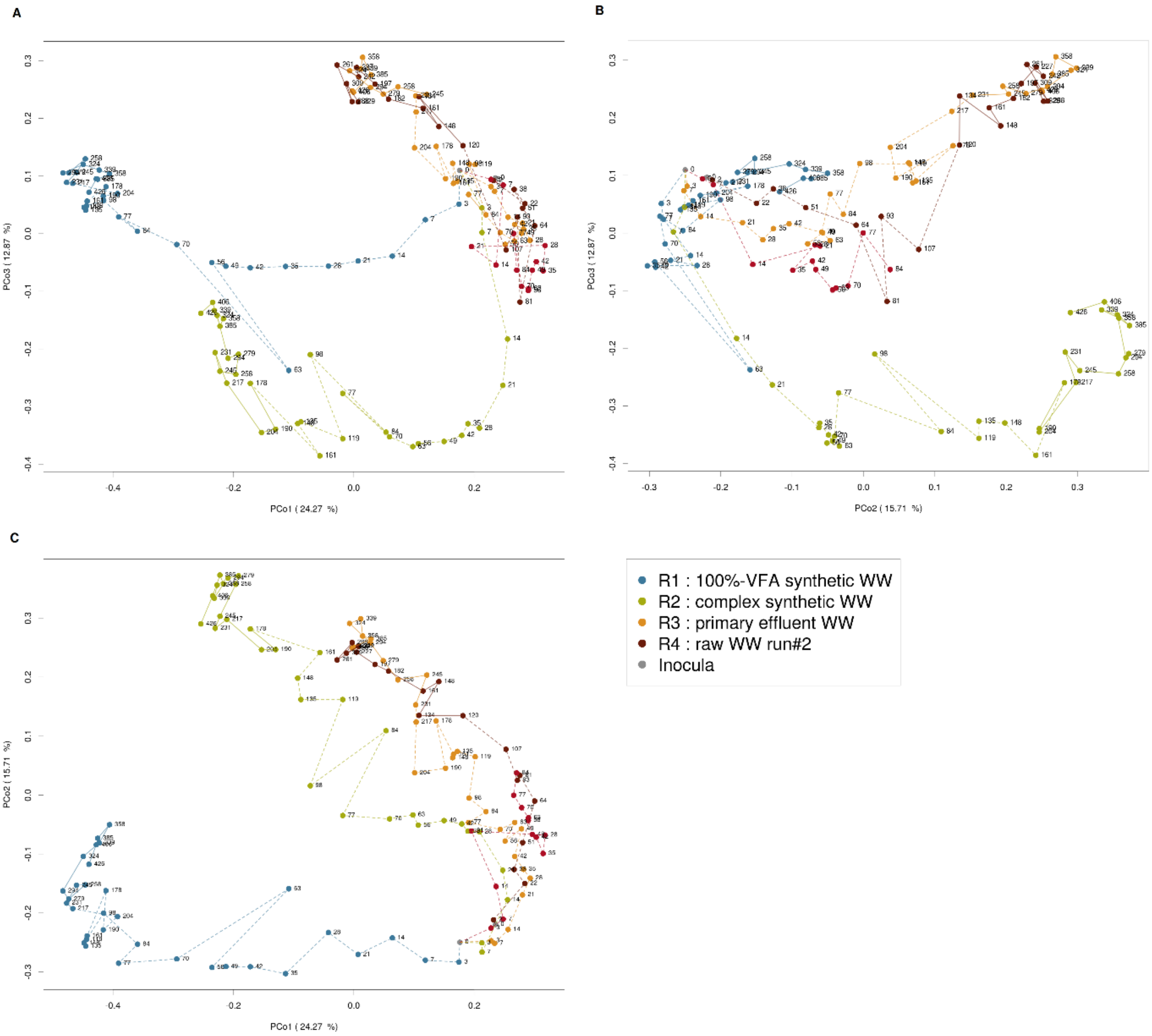

Figure S7: Principal component analysis of microbial communities structure evolution during the experiment, (**A**) on the 1st and 3rd axis (**B**) on the 2nd and 3rd axis and on the 1st and 2nd axis (**C**)

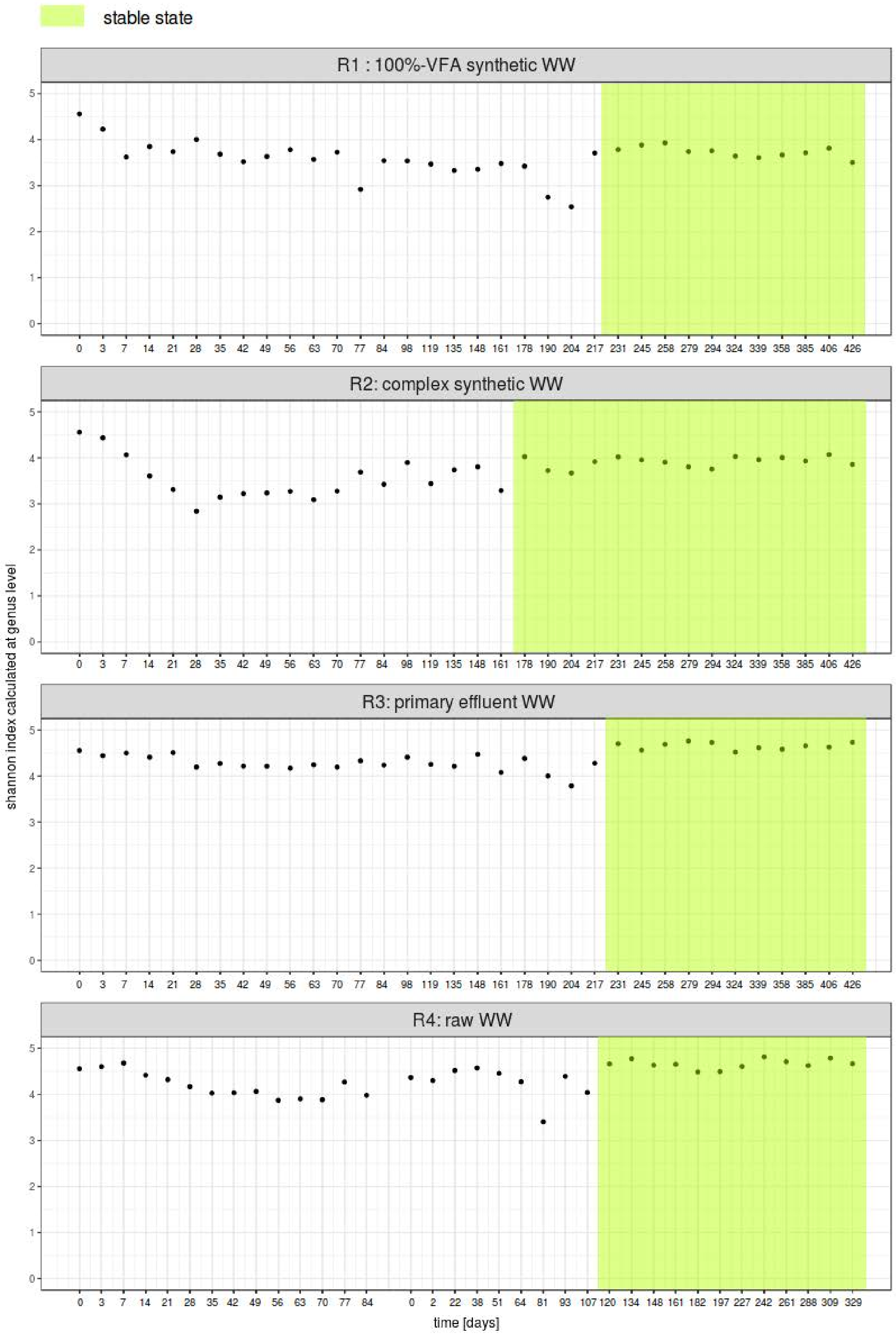

Figure S8: Evolution of the Shannon diversity index during the experiment in the four reactors. The values corresponding to the reactor treating raw WW (R4) are shown for both run#1 and run#2. The periods corresponding to the stable state of the bacterial communities are indicated in green.

Table S9: p-values of the t-test based on the null hypothesis that the mean relative abundance is the same in two different reactors at stable state for a selection of taxa. The complete table is in the file “S9_discriminant_and_p_value_t_tests.xlsx”.

Table S10: p-values of the t-test comparing the relative abundance of the main genera in the floccular and the granular fraction of the sludge.

Genus	R1	R2	R3	R4
480_2 (f)	1.8E-01	9.7E-02	4.8E-01	5.7E-01
Ca. Accumulibacter	9.8E-01	4.8E-04	8.8E-01	8.6E-01
Azoarcus	5.6E-01	8.5E-01	1.7E-01	4.9E-02
Ca. Competibacter	4.6E-02	9.0E-03	4.5E-01	2.8E-02
CPB_CS1	2.0E-01	4.0E-01	6.5E-01	2.1E-01
CYCU-0281	6.5E-01	4.4E-01	6.8E-01	9.7E-01
Flavobacterium	9.1E-01	1.2E-01	5.7E-02	4.4E-01
Kouleothrix	4.8E-01	6.3E-01	7.2E-02	4.3E-02
Micropruina	6.3E-02	3.0E-04	3.9E-03	6.1E-03
Nitrospira	2.2E-06	5.6E-02	5.3E-01	5.0E-08
P58	8.9E-01	9.4E-01	1.9E-01	8.3E-01
Rhodobacter	3.5E-04	3.0E-01	7.2E-01	3.4E-01
Runella	2.8E-02	3.3E-01	9.8E-01	4.9E-01
Saccharibacteria (p)	5.7E-02	2.8E-01	6.1E-01	2.6E-01
Tetrasphaera	7.1E-01	6.7E-01	6.3E-01	5.0E-01
Trichococcus	2.9E-03	5.9E-01	1.8E-01	3.9E-03
Xanthomonadaceae (f)	6.8E-01	2.2E-03	1.4E-01	7.8E-02
Zoogloea	1.1E-02	2.0E-04	3.8E-01	1.6E-01
Terrimonas	2.7E-04	5.8E-01	4.8E-01	3.1E-01
Thauera	3.1E-01	9.8E-01	6.5E-01	8.8E-06

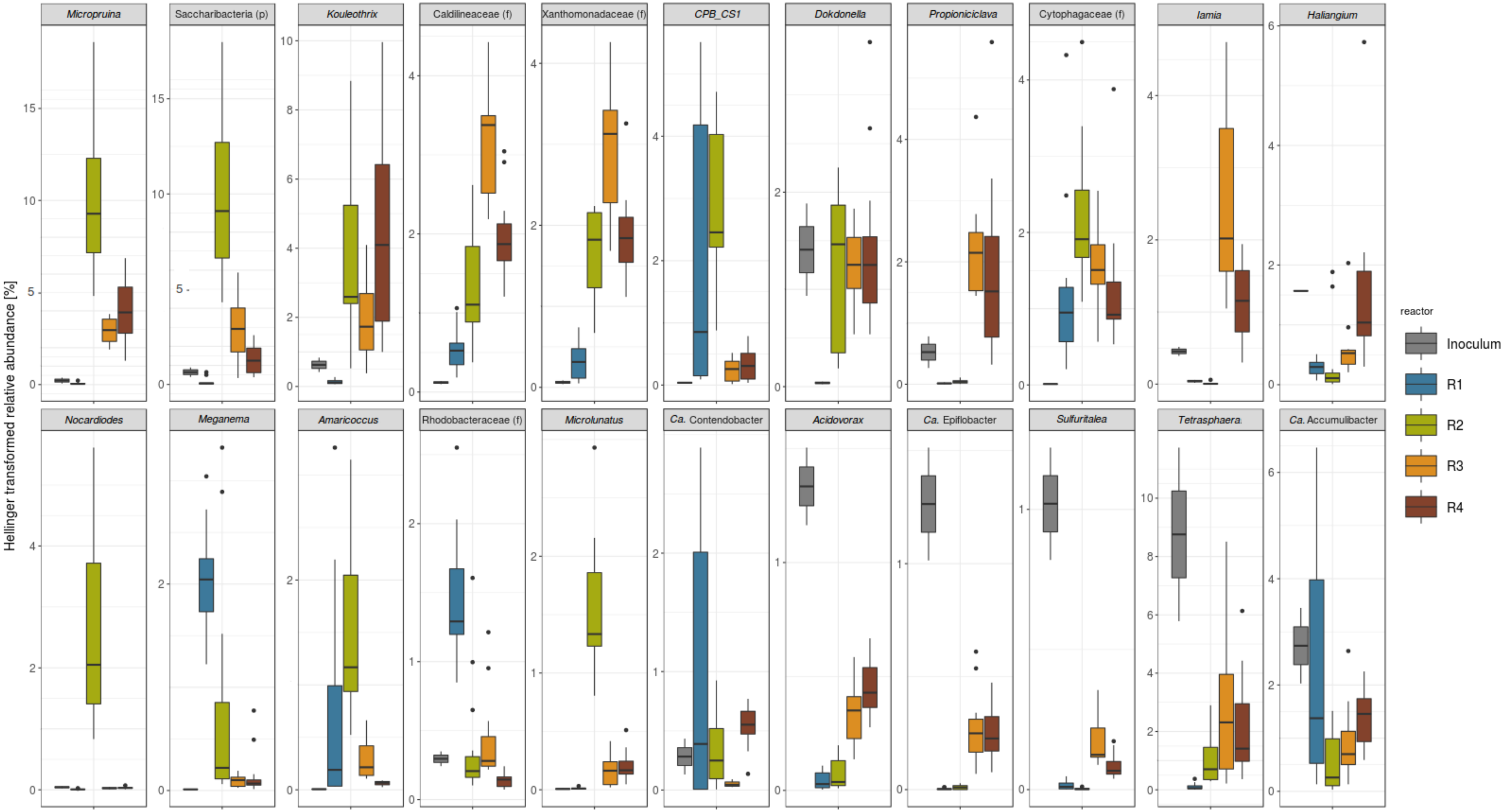

Figure S11: Boxplot of the Hellinger transformed abundance of the more discriminant genera (at least one p-value below 10 E-7 in the t-test), *Tetrasphaera* and *Ca.* Accumulibacter, in the four reactors and the inocula.

Table S12: Bray-Curtis distance matrices of the bacterial community compositions, the settling properties and size distribution of the sludge and its nutrient-removal performances at stable state. The three complete tables are in the file “S12_bray_distance_matrices_stable_state.xlsx”.

Table S13: Mantel test comparing the Bray-Curtis distance matrices of the microbial communities, the settling and size characteristics of the sludge and its nutrient-removal performance, at bacterial stable state.

Dataset	Mantel statistic	Significance
Microbial communities vs settling	0.6454	0.001
Microbial communities vs nutrientremoval	0.0470	0.195
Settling vs nutrient-removal	0.2138	0.009

Table S14: Average relative abundance of the bacterial taxa (at genus level) in the inoculum and the four reactors during stable state. The complete table is in the file "S14_genera_mean_abundance_at_stable_state.xlsx".

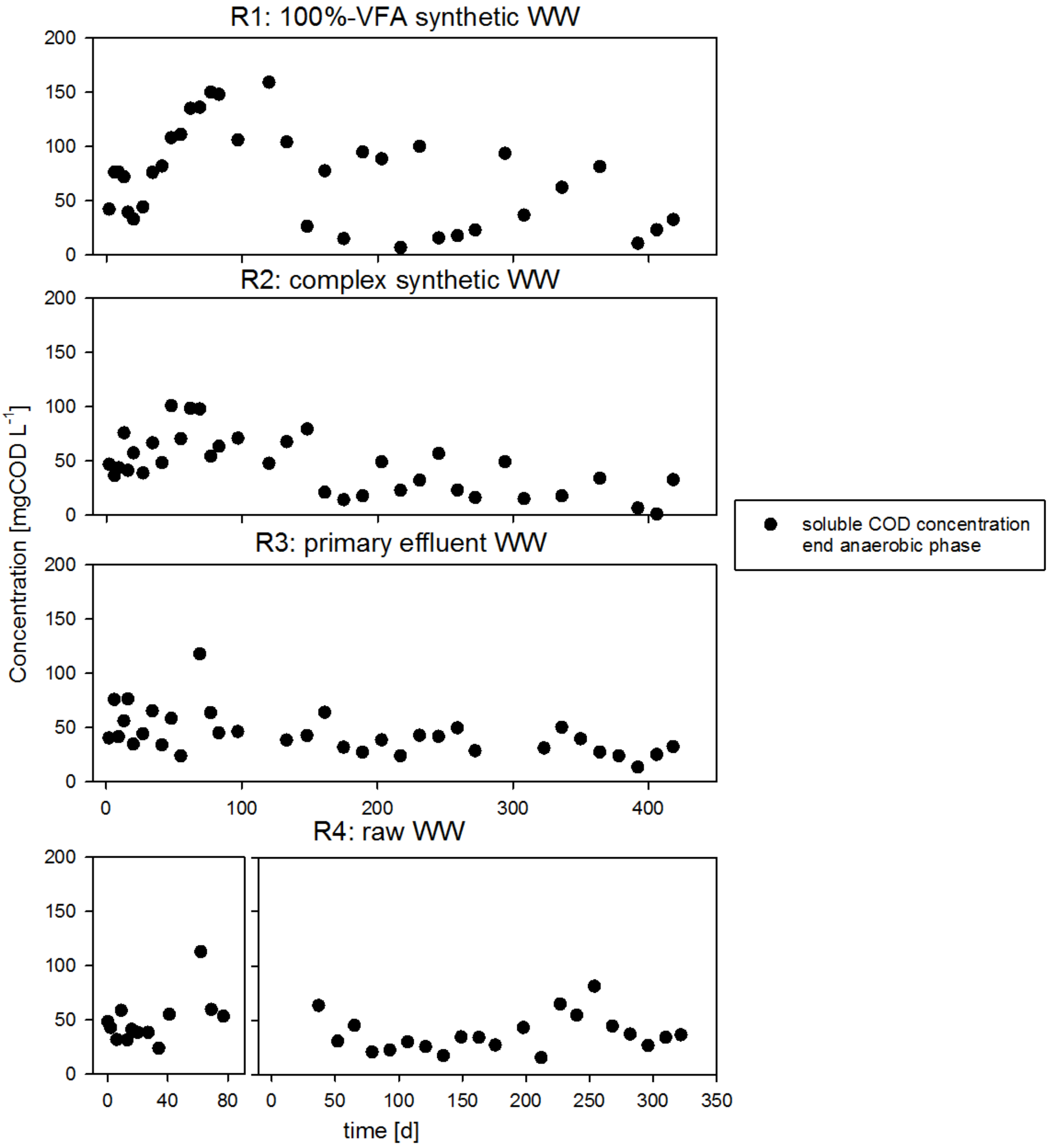

Figure S15: Soluble COD concentrations after the anaerobic phase of R1, R2, R3, R4 run#1 (row 4, left) and R4 run#1 (row 4, right).

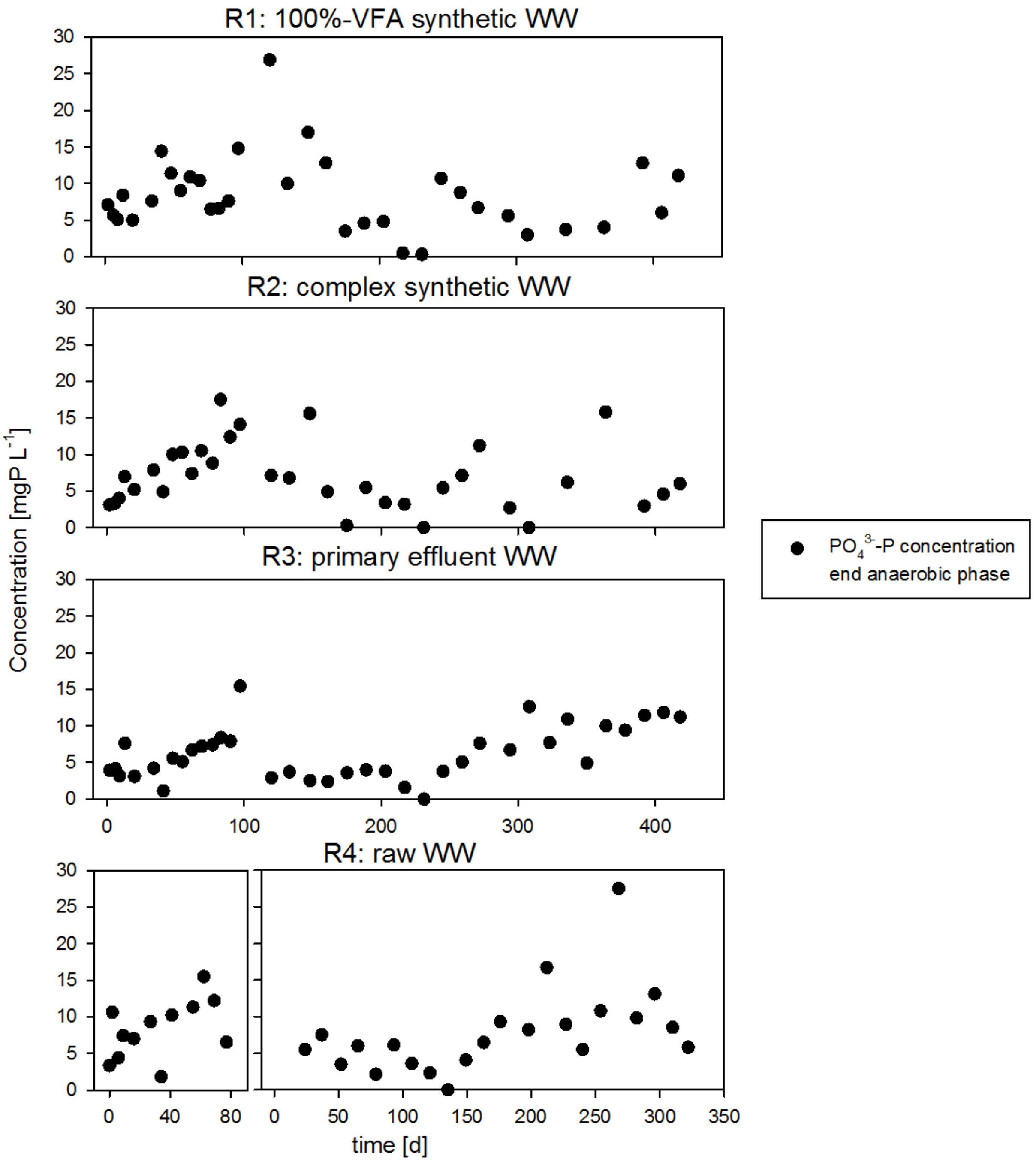

Figure S16: PO_4^{3-}-P concentrations after the anaerobic phase of R1, R2, R3, R4 run#1 (row 4, left) and R4 run#1 (row 4, right).

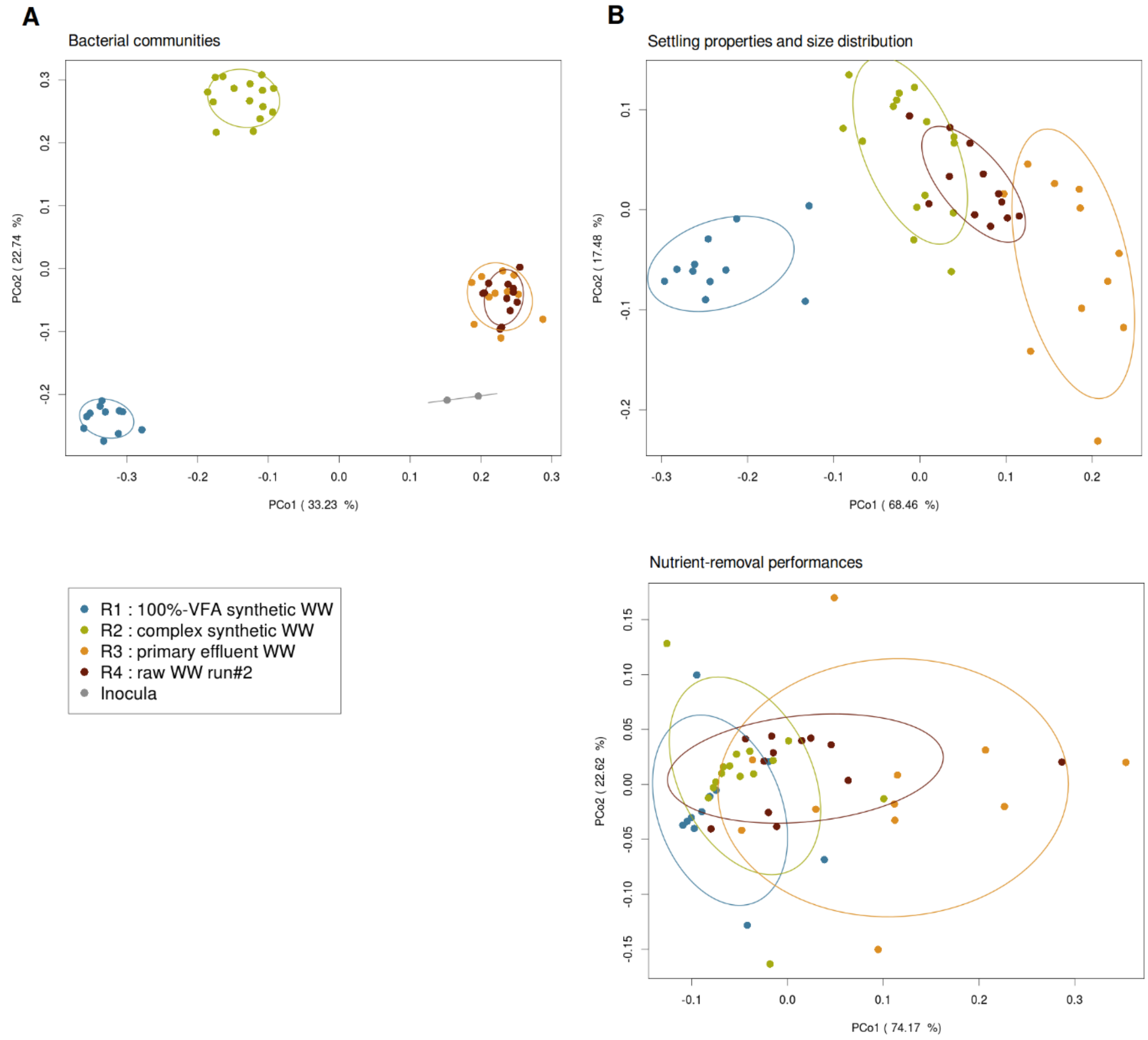

Figure S17: Principal component analysis based on the bray-curtis distance between the relative abundance of bacterial taxa (**A**), the granule size distributions and settling properties (**B**), and the nutrient-removal performances (**C**) in the four reactors during bacterial stable state. The ellipses indicate the 70% confidence interval for the data related to each reactor.
